# Internally consistent and fully unbiased multimodal MRI brain template construction from UK Biobank: Oxford-MM

**DOI:** 10.1162/imag_a_00361

**Published:** 2024-11-25

**Authors:** Christoph Arthofer, Stephen M. Smith, Gwenaëlle Douaud, Andreas Bartsch, Fidel Alfaro-Almagro, Jesper Andersson, Frederik J. Lange

**Affiliations:** Wellcome Centre for Integrative Neuroimaging, FMRIB, Nuffield Department of Clinical Neurosciences, University of Oxford, Oxford, United Kingdom; Department of Neuroradiology, University of Heidelberg, Heidelberg, Germany

**Keywords:** multimodal, template, age-dependent, UK Biobank, registration

## Abstract

Anatomical magnetic resonance imaging (MRI) templates of the brain are essentialto group-level analyses and image processing pipelines, as they provide areference space for spatial normalisation. While it has become common forstudies to acquire multimodal MRI data, many templates are still limited to onetype of modality, usually either scalar or tensor based. Aligning each modalityin isolation does not take full advantage of the available complementaryinformation, such as strong contrast between tissue types in structural images,or axonal organisation in the white matter in diffusion tensor images. Mostexisting strategies for multimodal template construction either do not use allmodalities of interest to inform the template construction process, or do notuse them in a unified framework. Here, we present multimodal, cross-sectionaltemplates constructed from UK Biobank data: the Oxford-MultiModal-1 (OMM-1)template and age-dependent templates for each year of life between 45 and 81years. All templates are fully unbiased to represent the average shape of thepopulations they were constructed from, and internally consistent throughjointly informing the template construction process with T1-weighted (T1),T2-weighted fluid-attenuated inversion recovery (T2-FLAIR), and diffusion tensorimaging (DTI) data. The OMM-1 template was constructed with a multiresolution,iterative approach using 240 individuals in the 50–55-year age range. Theage-dependent templates were estimated using a Gaussian process, which describesthe change in average brain shape with age in 37,330 individuals. All templatesshow excellent contrast and alignment within and between modalities. The globalbrain shape and size are not preconditioned on existing templates, althoughmaximal possible compatibility with MNI-152 space was maintained through rigidalignment. We showed benefits in registration accuracy across two datasets (UKBiobank and HCP), when using the OMM-1 as the template compared withFSL’s MNI-152 template, and found that the use of age-dependent templatesfurther improved accuracy to a small but detectable extent. All templates arepublicly available and can be used as a new reference space for uni- ormultimodal spatial alignment.

## 1 Introduction

Anatomical magnetic resonance imaging (MRI) templates of the brain aim to providerepresentative models of average shape and voxel signal intensity of the populationsfrom which they were constructed. They are essential for many different kinds ofneuroimaging analyses as they provide a common reference space for the spatialnormalisation of individual subjects using image registration methods. The resultingtransformations and derived measures, such as Jacobian determinant maps, betweeneach individual and a template, and the transformed images in template space, enablethe study of intra- and intergroup variability or agreement, unbiased groupcomparisons of within-subject longitudinal changes, and atlas-based segmentation ofregions of interest (ROIs) at subject level.

Template construction methods aim to find an average intensity and average shapetemplate, that is, the shape and intensity of the template should, on average, notbe more like any one individual than any other (seeSection 2.4.2for mathematical description). This is typically achievedthrough a series of steps to avoid bias in appearance or shape towards any singleindividual. The most commonly used method is based on an iterative framework (Guimond et al., 1998,^2000^), which was later extended into a multiresolutionapproach with a hierarchical processing structure (Fonov et al., 2011;Grabner et al.,2006). First, individual images are corrected for global (affine)misalignment using translation, rotation, scale, and shear, which allows for theconstruction of an initial average affine template. Each individual is theniteratively nonlinearly registered to the current template (starting with the affinetemplate in the first iteration), followed by spatial unbiasing of the warps, andresampling of the subject images. Finally, the average across the resampled imagesbecomes the new template and serves as the reference space for the next iteration.These steps are repeated until convergence, while warp resolution and image blurringare adjusted from coarse to fine.

Existing templates are often described as uni- or multimodal based on the number ofmodalities they comprise. An overview of some of the most commonly used and somemore recent templates can be found inTable1. In contrast to one modality in unimodal templates, multimodaltemplates aim to provide volumes of different, but anatomically corresponding,modalities. This notion of multimodality in most existing templates stems from thepost hoc availability of multiple modalities in template space, but generally doesnot refer to the modalities used during the template construction process. Drivingthis process with complementary information from different modalities of interest ishighly desirable since it can improve registration quality. For example, the axonalorganisation derived from diffusion imaging data can add valuable information aboutthe white matter, which would not be available from T1-weighted (T1) images only.Some existing templating methods use one modality to drive the construction, forexample, T1, and then apply the same deformation fields to all modalities ofinterest, for example, T2-weighted (T2) or diffusion tensor images (DTI) (Fonov et al., 2011;Gupta et al., 2016;Rohlfing et al., 2010). Others use modalities*derivedfrom*the modality of interest, for example, fractional anisotropy (FA)maps from DTI, to drive the construction and then transform the modality of interest(DTI) with the same transformations (Lv et al.,2022;Zhang et al., 2011).Estimating deformation fields based on a subset of modalities or surrogates andapplying the same deformation fields to all other modalities is not optimal. Thisstrategy can lead to unwanted biasing effects in the template, since not allmodalities contribute to the estimation of the deformation fields that are used forresampling and spatial unbiasing. This might not have a large impact when usingmodalities with similar information content, for example, when estimating a warpbased on T1 images and applying the same warp to T1 and T2 images. However, formodalities with different information content, it could introduce a spatial bias.For example, estimating deformation fields based on structural or diffusion-derivedscalar modalities, and applying them to diffusion tensors could lead to a bias inthe location or orientation of the diffusion data.

**Table 1:** Overview of existing unimodal and multimodal templates.

Template	Template modalities and maps	#Subjects	Mean age ± sd (min–max)	Ref.
ICBM MNI 305	**T1**	305 (66f/239m)	23.4 ± 4.1 (NA)	Evans et al. (1993)
ICBM 152 linear	**T1** , T2, PD	152 (66f/86m)	25.02 ± 4.9 (18–44)	Mazziotta et al. (1995)
ICBM 152 nonlinear 6th gen.	**T1**	152 (66f/86m)	25.02 ± 4.9 (18–44)	Grabner et al. (2006)
ICBM 2009a	**T1** , T2, PD, T2 relaxometry PVMs (GM, WM, CSF)	152 (66f/86m)	25.02 ± 4.9 (18–44)	Fonov et al. (2011)
ICBM 2009b	**T1** , T2, PD	152 (66f/86m)	25.02 ± 4.9 (18–44)	Fonov et al. (2011)
ICBM 2009c	**T1** , T2, PD PVMs (GM, WM, CSF)	152 (NA)	NA	Fonov et al. (2011)
ICBM 152 extended nonlinear	**T1** , T2, PD	152 (66f/86m)	25.02 ± 4.9 (18–44)	Fonov et al. (2011)
SRI24	**T1** , T2, PD, FA, MD, LD, mean DWI, PVMs (GM, WM, CSF), tissue labels, 2 CPMs	12 young (6f/6m) 12 elderly (6f/6m)	25.5 ± 4.34 (19–33)77.7 ± 4.9 (67–84)	Rohlfing et al. (2010)
Enhanced ICBM DT template	DTI, **PVMs (GM, WM, CSF) based on FA/trace map**	67 (40f/27m)	f: 27.2 ± 5.4 (20–39)m: 31.7 ± 5.6 (22–44)	Zhang et al. (2011)
Clinical DTI	**T1** , DTI	48 (NA)	NA	Gupta et al. (2016)
FOD template	FOD, **T1, T2, MD, FA, AFD, CX**	50 (25f/25m)	NA (22–35)	Lv et al. (2022)
MINT ABCD atlas	** T1, PVMs (GM, WM), 0 ^th^ , 2 ^nd^ order SHCs of restricted FOD 0 ^th^ order SHC of hindered & free water FODs **	BL 11140 (5353f/5787m) FU 7578 (3503f/4075m)	median: 9.9 (8.9–11)median: 11.9 (10.6–13.8)	Pecheva et al. (2022)
TBI template	**T1, DTI**	TBI 12 (5f/7m) HC 9 (3f/6m)	35 ± 12.1 (21–59) 36.2 ± 8.8 (23–46)	Avants et al. (2008)
HCP atlas	**T1, T2, DTI**	971 (520f/451m)	NA (22–35)	Irfanoglu et al. (2020)
MIITRA atlas	**T1, DTI**	202 (101f/101m)	80.56 ± 8.14 (65.2–94.9)	Wu et al. (2022)

**Modalities in bold**are used in the construction.

PD = proton density weighted, LD = longitudinaldiffusivity, DWI = diffusion-weighted imaging, PVMs =partial volume maps, CPMs = cortical parcellation maps, SHC= spherical harmonics coefficient, AFD = apparent fibredensity, CX = fibre complexity, FOD = fibre orientationdistribution, BL = baseline, FU = follow-up.

One fully unbiased multimodal (FUMM) template was constructed from individuals in theadolescent brain and cognitive development (ABCD) study. For this template, 11scalar modalities, including 3 structural modalities and 8 dMRI-derived modalitiesbut no DTI data were used as input to the Multimodal Image Normalisation Tool (MINT)(Pecheva et al., 2022). Another FUMMtemplating strategy for scalar and tensor modalities was applied in the constructionof the MIITRA template (Wu et al., 2022). Themethod alternates between registrations within each of the T1 and DTI modalities. Ineach iteration, deformation fields are estimated within one of the two modalitieswith a modality-specific registration method. The same transformations are appliedto data from both modalities in all iterations except the last, where the DTI dataundergo one more transformation that is not applied to the T1 images. A similariterative approach, involving multiple repeated registrations with the two methods,is required when spatially normalising individuals to the MIITRA template. Sinceboth modalities drive the template construction, the resulting templates are fullyunbiased. However, the use of two methods does not provide a unified and internallyconsistent framework. To the best of our knowledge, the only two methods that canaccommodate both scalar and tensor modalities, and, consequently, allow fullyunbiased and internally consistent template construction, are SymmetricNormalization for Multivariate Neuroanatomy (SyNMN) (Avants et al., 2008) and DR-TAMAS (Irfanoglu et al., 2016). SyNMN was applied in the construction of acombined T1 and DTI template to investigate traumatic brain injury (TBI) and laterin the construction of a template from arterial spin labelling, T2-weightedfluid-attenuated inversion recovery (T2-FLAIR), DTI, functional MRI (fMRI), T1 andT2 data (Tustison et al., 2015). The SyNMNtool and templates are not publicly available at the time of writing. DR-TAMAS hasbeen used for the construction of a DTI atlas (Irfanoglu et al., 2020) from the Human Connectome Project Young Adult(Van Essen et al., 2012) dataset(22–35-year age range). This DTI template also comprises T1 and T2 volumes,and all modalities were used to drive the registrations during the templateconstruction process. The atlas was constructed from 971 individuals and has goodlevels of detail and contrast (although not quite as good as might be hoped for,given the quality of the data and the number of subjects).

Most existing multimodal templates provide a single, cross-sectional average of brainshape and intensity from the subjects in a cohort. However, arguably, a templateshould also be similar to a given population under investigation to reduce theamount of deformation required when aligning individuals to it. The main factorcontributing to morphological variability in large datasets is the subjects’age range. As datasets become larger in size and the subjects’ age rangewithin datasets increases, it becomes more difficult to capture the age-relatedincrease in brain shape variability in a single template. Spatiotemporal, orage-dependent templates (ADTs), for subpopulations with smaller age ranges, canprovide more similar reference spaces. Several construction methods based ondiscrete bins (Fillmore et al., 2015), kernelregression (Davis et al., 2007;Serag et al., 2012), and neural network-basedarchitectures (Dalca et al., 2019;Wilms et al., 2020;Xia et al., 2019;Zhao etal., 2019) have been described in the literature. However, these havebeen mainly used for unimodal ADT construction and, to the best of our knowledge, donot publicly provide multimodal templates with scalar and tensor modalities forgeneral use.

### 1.1 Summary of our work

The main contributions of our work include the construction of a cross-sectional,internally consistent and fully unbiased multimodal, whole-head template, theOxford-MultiModal-1 (OMM-1), and the development of a modelling andprediction-based approach, which was applied in the construction of multimodal,average-shape ADTs.

The former was obtained from 240 UK Biobank (UKB) individuals (50–55years, 50% females) with the iterative approach described inFonov et al. (2011). First, we constructed anunbiased affine template, which was refined from coarse to fine by iteratingthrough nonlinear registrations, and unbiasing, warping, and averaging steps.The ADTs were obtained by nonlinearly registering 37,330 UKB individuals(45–82 years) to OMM-1 and using the acquired deformation fields andcorresponding individuals’ ages to model the change in average brainshape with age using a Gaussian process (GP). Finally, the trained model allowedus to predict and apply a mean deformation field for each year of age to deriveage-dependent templates from the initial 240 UKB individuals.

The OMM-1 and its associated ADTs provide anatomically corresponding scalar (T1and T2-FLAIR) and tensor (DTI) volumes. These same modalities were used to drivethe construction process by simultaneously informing the nonlinearregistrations. These registrations were performed with FSL’s MultiModalRegistration Framework (MMORF) (Lange,Ashburner, et al., 2020;Lange,Smith, et al., 2020;Lange et al.,2024), which estimates a single warp by optimising over an arbitrarynumber of scalar and tensor input modalities. This ensures internal consistencyby avoiding the need to use different registration methods for differentmodalities, and full unbiasing of all volumes with respect to all modalities ofinterest. Our template construction pipeline provides a unified framework thatcan easily be extended or adjusted to other scalar or tensor modalities ofinterest. Since the OMM-1 is unbiased with respect to the UKB subjects fromwhich it was created, its size and shape differ from the most commonly usedtemplate, the MNI 152 (in its various revisions), as MNI 152 templates are notvery close to representing the size of the average adult brain. However, theOMM-1 was rigidly (six degrees of freedom) aligned to MNI space, andtransformations between the two templates are provided, to aid compatibilitywhen switching between them. Finally, we have investigated the benefits of usingour multimodal templating framework for spatial normalisation in age-diversepopulations of two datasets.

## 2 Methods

### 2.1 Data

In this work we used scalar- and tensor-valued, nondefaced brain MRI data fromUKB (Miller et al., 2016), one of thelargest prospective epidemiological studies to date, which aims to acquiremultimodal MR imaging data from 100,000 participants.

Imaging data from three MRI modalities including T1, T2-FLAIR, and DTI were usedfor template construction. T1 provides information about the basic anatomicalstructure of the brain and shows strong contrast between the main tissue classes(grey and white matter, and cerebrospinal fluid). It is acquired as part of mostimaging studies and has become the core modality of choice for existing adulthuman templates. T2-FLAIR was included as a second structural modality due toits enhanced contrast of subcortical grey matter regions, such as the striatum,pallidum, substantia nigra, red nucleus, and dentate nucleus, of the olfactorybulbs, but also between normal appearing white matter and white matterhyperintensities. Diffusion MRI provides information about the properties of thelocal tissue microstructure and white matter tract structure. It makes itpossible to estimate a diffusion tensor for each voxel (Basser et al., 1994) that adds information about theaxonal organisation and the preferred directions of diffusion. We decided to usenondefaced T1 and T2-FLAIR data to construct a template that is sharp and clearin both intra- and extracranial regions and, hence, may be useful for a varietyof applications.

All imaging data were collected at one of three UKB sites using identical 3TSiemens Skyra scanners running VD13 and a standard Siemens 32-channel receiverhead coil. A brief overview of the parameters used to acquire T1, T2-FLAIR, anddMRI can be found inTable 2. For adetailed description of the acquisition protocol in the UKB brain imaging study,we refer the reader toMiller et al.(2016).

**Table 2:** UK Biobank brain MRI acquisition parameters for T1, T2-FLAIR, and dMRIfromMiller et al. (2016).

Modality	Voxel size, Matrix	Key parameters
T1	1.0 × 1.0 × 1.0 mm, 208 × 256 × 256	3D MPRAGE, sagittal, R = 2, TI/TR = 880/2000 ms
T2-FLAIR	1.05 × 1.0 × 1.0 mm, 192 × 256 × 256	FLAIR, 3D SPACE, sagittal, R = 2, PF 7/8, fat sat, TI/TR = 1800/5000 ms, elliptical
dMRI	2.0 × 2.0 × 2.0 mm, 104 × 104 × 72	MB = 3, R = 1, TE/TR = 92/3600 ms, PF 6/8, fat sat, b = 0 s/mm ^2^ (5 × + 3 × phase-encoding-reversed), b = 1000 s/mm ^2^ (50×) b = 2000 s/mm ^2^ (50 × )

R = in-plane acceleration factor, MB = multibandfactor, PF = partial Fourier.

The OMM-1 was constructed from 240 individuals uniformly and randomly sampledfrom the 50–55-year age range (40 individuals per year, 50% female).Using the ethnic background categories provided by UKB (data-field 21000), thesample is made up of 224 White, 3 Chinese, 1 Black or Black British, 6 Asian orAsian British, 1 Mixed, 3 other ethnic groups, and 2 not available. The size ofthe sample was informed by previous investigations on the Human ConnectomeProject dataset, whereYang et al. (2020)have shown that sample sizes larger than 200 individuals are associated withonly small changes to the final templates. We selected individuals from theyounger end of the UKB age range that provided sufficient data for uniformsampling. This minimises the appearance of ageing-related features and,therefore, maximises the utility of the template when applied to studiesinvolving younger subjects.

**Fig. 1.**
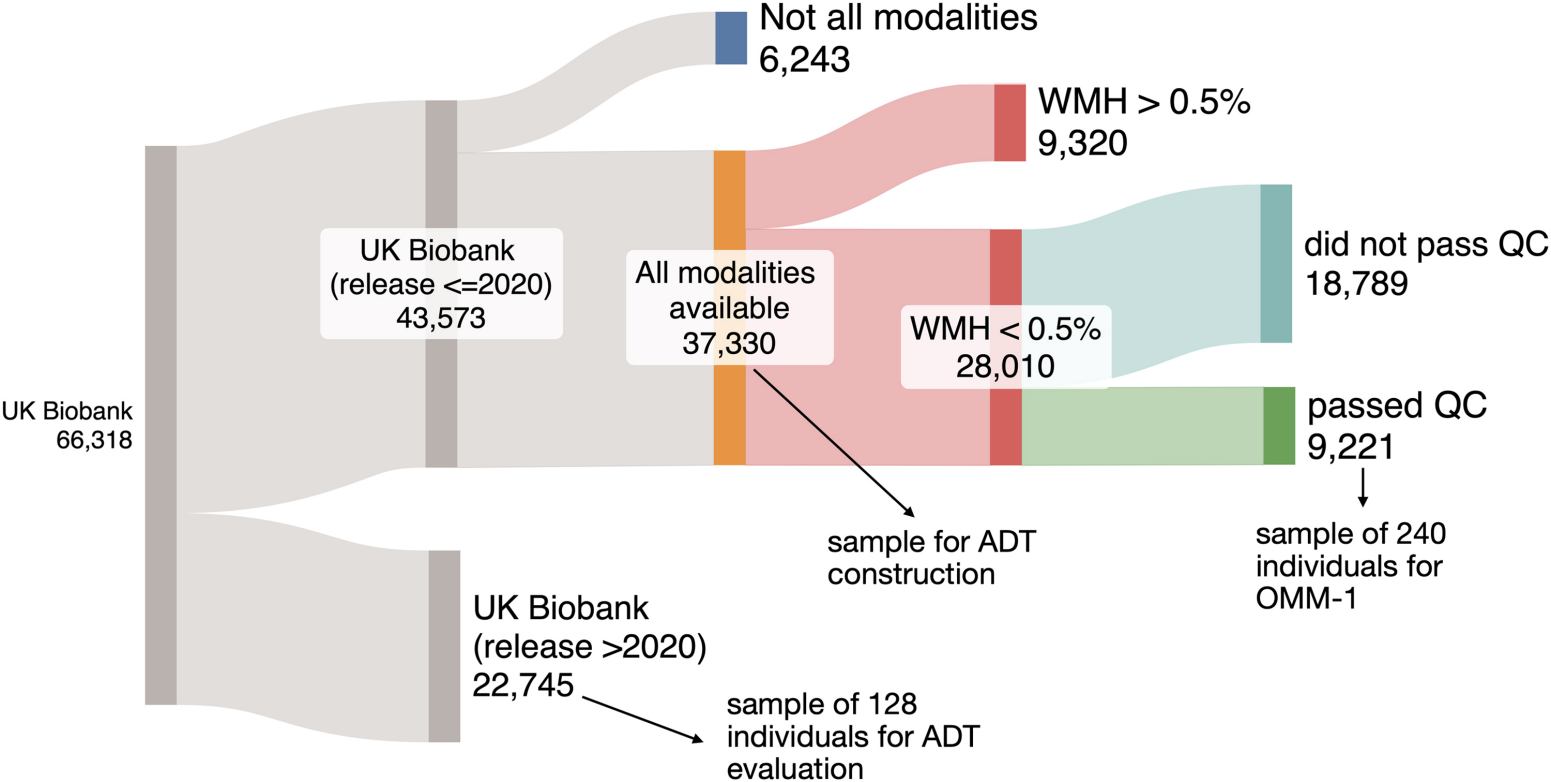
Overview of the strict OMM-formation QC filtering, which was applied tothe initial UKB dataset and the corresponding number of individualsremaining. WMH = white matter hyperintensities.

All UKB images went through the manual and automated quality control (QC)pipeline described inAlfaro-Almagro et al.(2018). Although badly corrupted images are excluded by thispipeline, several additional criteria for subjects to be considered in ourrandom sample were defined. In this work we only used images acquired at theindividuals’ first scanning appointments; repeat scans were excluded.Figure 1illustrates how we arrived atthe subject numbers that the samples were taken from after strict OMM-formationQC. The requirements included the availability of all three modalities, lessthan 0.5% of the total brain volume containing white matter hyperintensities(WMH), and small alignment discrepancies. Alignment discrepancy measurements hadbeen calculated as the correlation ratio between registered within-subjectmodalities by the QC pipeline and are available as QC imaging-derived phenotypes(IDPs) for all three modalities. Extreme scores are potential indicators forpoor alignment, or the presence of artefacts or outliers. In particular, we usedIDPs that describe the discrepancies between an individual’s T1structural image and the MNI 152 6th gen. (Grabner et al., 2006) after nonlinear alignment, and between theT2-FLAIR and the corresponding T1 image, and the dMRI and the corresponding T1image after linear alignment. Thresholds of 0.5 for the T1 and T2-FLAIR and 0.6for the dMRI discrepancies were applied, to allow for a large enough sample ofsubjects from the selected age groups.

For the construction of the age-dependent templates, images from 37,330 (age45–82 years) individuals, which had T1, T2-FLAIR, and dMRI data, wereused. Given this large sample, the image quality at the individual level isexpected to have less impact on the final average templates for age modellingcompared with the smaller sample used for the OMM-1. Therefore, no furtherselection criteria were applied. Note, however, that systematic differences inintensity with changes in age (such as the appearance of WMHs in many subjectsin the same region of the brain) would affect the ADT-warps in thoseregions.

Held-out data from two datasets were used for the validation, including a morerecent UKB release and the Human Connectome Project Young Adult (HCP-YA) dataset(Hodge et al., 2016). The UKB datawent through the same preprocessing pipeline described below. No furtherselection criteria were applied, to avoid any potential bias in the comparisonof the templates.

### 2.2 Ethics

Written informed consent was obtained from all UK Biobank participants. Theethical procedures (http://www.ukbiobank.ac.uk/ethics) for the UK Biobank to obtain andshare the data were approved by the North West Multi-centre Research EthicsCommittee (MREC).

The human imaging data used in the validation part of this work are part of theopen access Human Connectome Project Young Adult dataset. Written informedconsent to disseminate the data was obtained from all participants by the HCP-YA(Elam et al., 2021).

### 2.3 Data preprocessing

We used both minimally processed and preprocessed UKB imaging data. The T1,T2-FLAIR, and dMRI volumes of the former are gradient-distortion corrected, andthe T1 and T2-FLAIR volumes are not defaced, that is, they include parts of theneck, nose, and mouth. The latter had been preprocessed with the standardpipeline described inAlfaro-Almagro et al.(2018), which, in addition to gradient-distortion correction,includes defacing, cropping, brain extraction through atlas-based maskpropagation, and intensity inhomogeneity correction of T1 and T2-FLAIR images.Brain-extracted T2-FLAIR and dMRI images are rigidly coregistered to thecorresponding individual’s T1 reference space using the B0s as the movingimage and boundary-based registration (Greve& Fischl, 2009) as the cost function in FSL’s FLIRT(Jenkinson & Smith, 2001).dMRI data are corrected for susceptibility and eddy current distortion, as wellas head motion, using FSL’s topup (Andersson et al., 2003) and eddy (Andersson & Sotiropoulos, 2016;Andersson et al., 2016) before fitting the diffusiontensors (Basser et al., 1994) on the b= 1000 images (50 directions) with FSL’s DTIFIT.^*^This standardpreprocessing pipeline was extended for the template construction pipeline. Thebinary brain masks in individual dMRI spaces were slightly reduced in size bysmoothing with an un-normalised mean filter (3 x 3 x 3 kernel size, to create asmooth transition between brain and background), thresholding at 0.9, anderoding by one voxel. These new binary masks were used to reduce the impact ofnoisy DTI voxels at the border of and outside the brain during nonlinearregistrations. Bias fields created with FSL’s FAST were transformed fromeach individual’s reference spaces to their nondefaced T1 and T2-FLAIRnative spaces and used to correct for intensity inhomogeneity in the brain.High-intensity values of the scalp in T1 images were smoothly clamped with acustom function (seeAppendix A.1) toavoid negative effects on the nonlinear registrations during templateconstruction. We did not perform any resampling with the transformationsestimated between modalities to avoid the accumulation of interpolationerrors.

In the rest of this manuscript, we will use the following notation: the set ofimagesR,where each individualnfrom the set of*N*subjects has three modalitiesm∈{T1,T2−FLAIR,DTI}is defined as

$$R={\left\{R_{n}={\left\{R_{n}^{m}\right\}}_{m\in \left\{T1,\;T2-FLAIR,\;DTI\right\}}\right\}}_{n=1\;\;...\;N}.$$ (1)

### 2.4 Template construction

Our multimodal template and age-dependent template construction pipeline consistof three main parts.

**Fig. 2.**
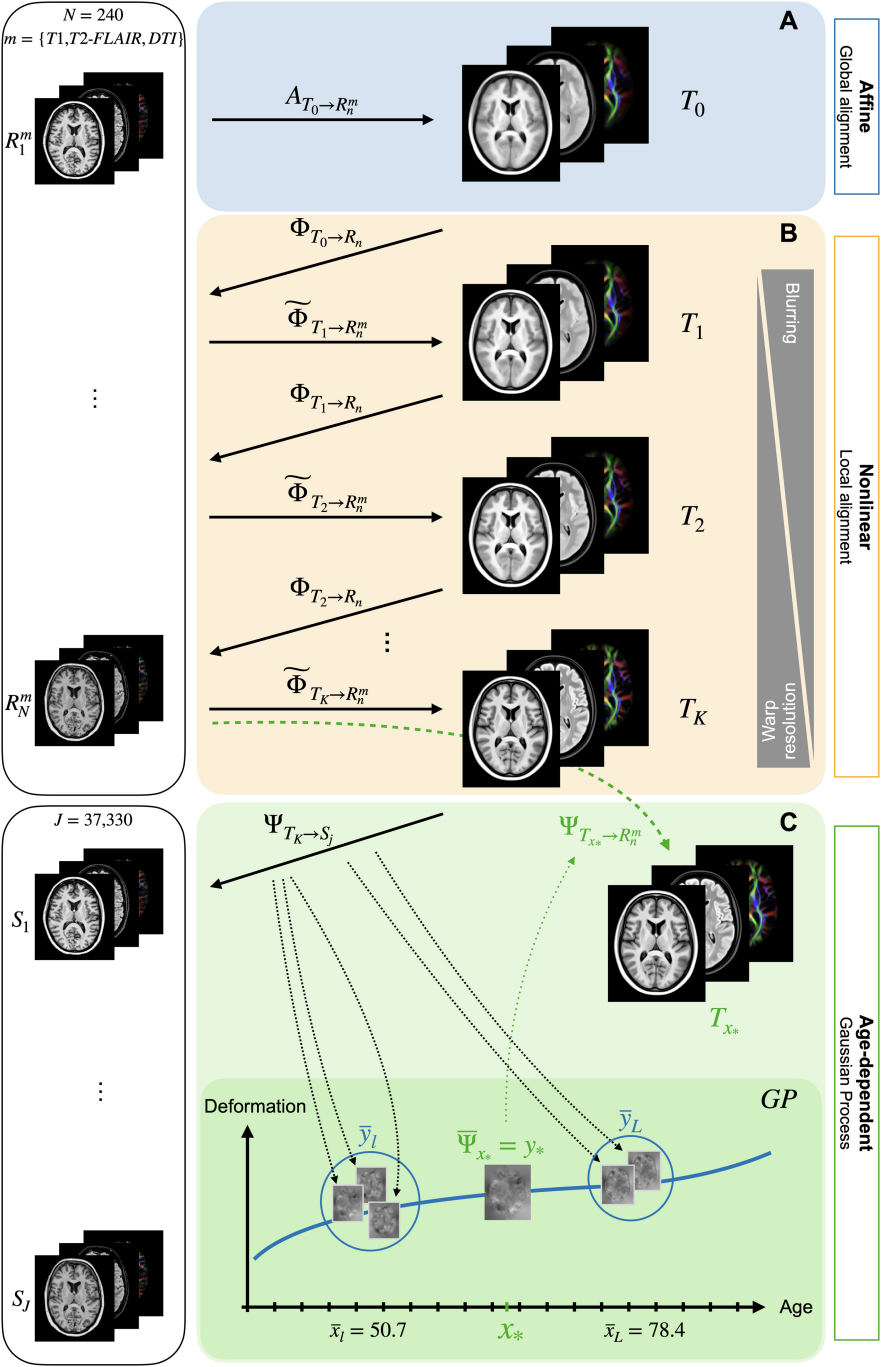
Scalar and tensor-based modalities from 240 UKB individuals were used toconstruct (A) the unbiased affine template by correcting for globalmisalignment and (B) the final nonlinear OMM-1 template by iteratingthrough the hierarchical optimisation approach. (C) Age-dependenttemplates were derived from the predictions of a Gaussian process modeltrained on ages and warps to OMM-1 space from 37,330 UKBindividuals.

First, an unbiased affine template was constructed by correcting for global(affine) misalignment between individuals (Fig.2A;Section 2.4.1), which wasthen rigidly aligned to MNI space (Grabner etal., 2006).

Second, this affine template was used to initialise a nonlinear, hierarchical,multiresolution templating approach (Fonov etal., 2011), which iterated through registration, unbiasing,transformation, and averaging steps (Fig.2B;Section 2.4.2). The finalnonlinear template, the OMM-1, represents the average shape and intensity of the240 individuals on which it is based.

Third, the OMM-1 was used as a template to spatially normalise 37,330 individualsfrom the UKB imaging cohort, resulting in one deformation field for each subject(Fig. 2C;Section 2.4.3). A Gaussian process (GP) was used to modelthe morphological differences captured by these deformation fields as a functionof age. After fitting the model, a mean deformation field was predicted forevery year of age between 45 and 81 years, and used to generate thecorresponding age-dependent template (ADT). In the following sections, we willdiscuss each of these steps in detail.

#### 2.4.1 Affine template construction

An initial affine template was constructed from the preprocessed,brain-extracted$R^{T1}$images. One subject was randomly selected as a referencespace and the remaining subjects were affinely registered to this referencewith 12 degrees of freedom (DOF). To avoid the introduction of a biastowards the brain geometry of the reference individual, the transformationfrom each individual’s space to the midspace of all subjects wascalculated using FSL’smidtransfunction. This function can take as input a set of affinetransforms, that is, one transform between each individual and thereference, and provide as output one affine transform between eachindividual and the midspace of all individuals. We performed preliminarytests with different subjects as an initial reference, and confirmed that wedid not find any difference in the final results. These estimatedtransformations were used to resample the corresponding brain-extracted$R^{T1}$images into the unbiased space. The first affine templatewas created by calculating voxel-wise the median over the resampled images,which provides a sharper group average compared with taking the mean at thisearly stage (Fig. 2A) and was found toimprove registration performance in the subsequent iterations.

This initial template was rigidly (6 DOF) aligned to the space of theasymmetric version of the nonlinear 6th gen. ICBM 152 template (MNI 152)(Grabner et al., 2006) includedin FSL, to maximise similarity between the spaces while avoiding shearingand scaling effects. The template after alignment is annotated as$T_{0}$.The final set of linear transformations$A_{n\text{​}}=\text{​}\left\{A_{T_{0}\to R_{n}^{T1}},A_{T_{0}\to R_{n}^{T2-FLAIR}},A_{T_{0}\to R_{n}^{DTI}}\right\}$for each individual was created by concatenating the corresponding rigidtransformation from each modality’s native space to T1 referencespace, the affine transformation from the T1 reference space to the unbiasedtemplate space, and the rigid transformation to the space of the newtemplate$T_{0}$.Nondefaced images were transformed from their native spaces to$T_{0}$by applying the corresponding concatenated transformations using splineinterpolation. Additionally, binary T1 and DTI brain masks were resampledwith the same transformations using trilinear interpolation resulting incontinuous-valued weighting masks. In contrast to binary masks, we willrefer to these nonbinary masks as soft masks throughout the manuscript.

The voxel-wise median of the resampled images for each of the modalitiesprovided the final affine template with three volumes$T_{0}=\left\{T_{0}^{T1},\;\;T_{0}^{T2-FLAIR},\;\;\;T_{0}^{DTI}\right\}$.Similarly, mean T1 and DTI soft masks were created from the individual softmasks in template space.

#### 2.4.2 Nonlinear template construction

As we have stated previously, it is desirable that a template not be biasedtowards any particular individual (or subset of individuals) in thepopulation from which it is constructed. By biased, we mean that thetemplate should not, on average, be more like any one subject than anyother. There are two ways in which a template might appear more similar toan individual: in its shape, and in its appearance—where appearancerefers to the voxel intensities. Consequently, a template may exhibit eithera shape bias, an appearance bias, or both, unless care is taken to avoidthis.

Shape (spatial) bias can be avoided by ensuring that, following registrationto the template, the average displacement from the template to eachindividual is minimised across the population. Appearance (intensity) biascan be avoided by ensuring that, following registration to the template, theaverage image dissimilarity metric used to drive the registration isminimised across the population. Dissimilarity metrics commonly used byregistrations methods include mean squared difference, cross-correlation,and mutual information.

Fonov et al. (2011)formalised thisconcept as finding the templateTthat simultaneously minimisesEquations 2and3, which address spatial and intensity bias, respectively. Theformer (Eq. 2) minimises themagnitude of the nonlinear deformations$\Phi_{T\to R_{n}}$required to warp the templateTto each subject$R_{n}$,and the latter (Eq. 3)minimises the mean squared intensity difference between the templateTand each warped subject$\Phi_{T\to R_{n}}(T)$. Note thatEquation 3is specific to our case where MMORF optimises animage dissimilarity metric that is a version of the sum of squareddifferences, and would differ if, for example, cross-correlation was usedinstead.

$$\underset{T}{\text{arg min}}\left[\sum_{n=1}^{N}{\left|\Phi_{T\to R_{n}}\right|}^{2}\right]$$ (2)

$$\underset{T}{\text{arg min}}\left[\sum_{n=1}^{N}{\left(T-\Phi_{T\to R_{n}}(T)\right)}^{2}\right].$$ (3)

In practice, these two steps are interleaved at each of multiple iterations.In iterationk,Equation 2is minimisedby “undoing” (inverting and applying) the average across allnonlinear deformations$\Phi_{T_{k}\to R_{n}}$required to warp the template$T_{k}$to each subject$R_{n}$,andEquation 3is minimisedby simple voxel-wise averaging of the warped intensities$\Phi_{T_{k}\to R_{n}}\left(T_{k}\right)$across all subjects.

Given this understanding of unbiasing at each stage/iteration of the templateconstruction pipeline, the optimal, unbiased, nonlinear OMM-1 templateTwas constructed by iterating over the following three steps (Fig. 2B).

1. Deformation fields$\Phi_{T_{k}\to R_{n}}$are estimated by nonlinearly registering each individual to thetemplate from the previous iteration$T_{k-1}$, with the affine template$T_{0}$being used as reference space for the first iteration. Registrationswere performed with MMORF (Lange etal., 2024) and were informed with both scalar modalities(T1, T2-FLAIR) and the tensor-valued modality (DTI) from individualand reference space. MMORF optimises the following total costfunction.
$$C_{TOT}\;=\lambda_{T1}C_{T1}\;+\lambda_{T2-FLAIR}C_{T2-FLAIR}\text{​}+\lambda_{DTI}C_{DTI}\text{​}+\lambda_{REG}C_{REG}$$ (4)
  with all modalities contributing equally in the optimisationprocedure (i.e.,$\lambda_{T1}=\lambda_{T2-FLAIR}=\lambda_{DTI}=1$). The mean squared error is calculated betweenscalar images, and the mean squared Frobenius norm is used as a costfunction for tensors. MMORF internally rescales the cost functionfor each image pair, that is, for each modality, to the same(arbitrary) value before every optimisation step duringregistration. This allows the image cost functionλvalues, i.e., the weights, to have approximately equivalent effectsfor a given value regardless of modality or image intensityscaling.
  Registrations were initialised with the corresponding lineartransformations$A_{n}$estimated during the construction of the affine template. Note thatnonbrain-extracted individual images and templates were used for thescalar channels, which poses additional challenges. Inclusion of theskull can negatively affect registration quality in nearby corticalregions, and the face and neck have larger anatomical and positionalvariability compared with the brain. To reduce the potential impactof extracranial tissue on the deformations close to the brain, andimprove registration quality in the face and neck, different levelsof relative regularisation were imposed on intra- and extracranialregions. Larger weights were given to regions inside the brain, thatis, reducing the relative level of regularisation to allow for moreaggressive deformations, and smaller weights were given to regionsoutside the brain, that is, increasing the relative level ofregularisation to constrain the deformations. Theintra-to-extracranial weight ratio was 8-to-1, which was achievedthrough modulation of the soft T1 brain mask in template space.Steps included thresholding at 0.1 and binarising, followed byrescaling the values through a series of arithmetic operations(multiplication by 7, addition of 1), while ensuring that the meanintensity value of the whole 3D volume was equal to 1(*-inm*flag in*fslmaths*).Similarly, a weighted average DTI soft mask with smoothly decreasingweights at the edge of brain tissue in reference space and erodedbinary DTI brain masks in individuals’ native spaces wereused to reduce the potential negative effect of poor/noisy tensorfitting around brain boundaries that are often seen in DTI. Thesewere created following the same steps described inSection 2.4.1. No masks wererequired for T2-FLAIR since tissue outside the brain already appearsdark and does not strongly drive the registration relative to braintissue.
2. The average deformation field${\bar{\Phi }}_{T_{k}}$was calculated with
  $${\bar{\Phi }}_{T_{k}}=\frac{1}{N}\sum_{i=1}^{N}\Phi_{T_{k}\to R_{n}}$$ (5)
  and used to spatially unbias the template. This unbiasing step wasperformed by composing its inverse${\bar{\Phi }}_{T_{k}}^{-1}$with each individual deformation field and thecorresponding rigid and affine transformations:
  $${\tilde{\Phi }}_{T_{k}\to R_{n}^{m}}={\bar{\Phi }}_{T_{k}}^{-1}\circ \Phi_{T_{k}\to R_{n}^{m}}\circ A_{T_{0}\to R_{n}^{m}.}$$ (6)
  Individuals’ modalities in their respective native spaces wereresampled to the new unbiased template space in one step by applying${\tilde{\Phi }}_{T_{k}\to R_{n}^{m}}$with spline interpolation. Tensors were reoriented with FSL’svecregtool, which uses the preservation of principaldirections algorithm (Alexander etal., 2001). Binary T1 and DTI brain masks were resampledwith trilinear interpolation using the same transformations.
3. New mean soft masks and a new template$T_{k}$were created in unbiased space by taking the average over theresampled images for each modality. This new unbiased template withits three volumes served as a reference space in the next iterationk+1. Since the scalar images are not quantitative,they were intensity normalised to a nonbackground mean of 1000.Tensors are quantitative and, therefore, no intensity normalisationwas necessary. Tensor averaging was performed in the log-Euclideandomain as this better preserves the degree of anisotropy present inthe original images and avoids tensor swelling when compared withsimple Euclidean averaging (Arsigny,Fillard, et al., 2006). Regarding averaging, the maskswere only used during the registration step, and the averaging wasperformed without masking. However (for the DTI channel inparticular), there are some voxels towards the edges of the brainthat do not have valid values for all individuals—that isthey may be zero or have negative determinants. In these voxels, theaverage is taken only across subjects with valid data at thatvoxel.

We performed a total ofK=18iterations, allowing for coarse to fine improvements, withthree iterations at each of six hierarchical levels. A large grid spacing of32 mm and a blurring kernel of 8 mm FWHM were used for the MMORFregistrations (step 1) in the three iterations at the first hierarchicallevel. These parameter values were halved for each level, down to 1 mm and0.25 mm (respectively) at the last hierarchical level. An overview of theMMORF registration parameters can be found inAppendix Table A.1.

We also created a female and a male template by averaging the warps of the120 females and the 120 males, and applying their inverses to the OMM-1template. More details can be found inAppendix A.7.

#### 2.4.3 Age-dependent template construction

UKB individuals$S={\left\{S_{j}\right\}}_{j=1...J}$(J=37,330) from the 45–82-year age range were first affinelyand then nonlinearly registered to OMM-1. Similar to the previous nonlinearregistrations, we used MMORF with all three MRI modalities (Fig. 2C) and the registration parametersdescribed inAppendix A.3. Due tocomputational requirements associated with the large number of individuals,the highest warp resolution was set to 2 mm instead of the 1 mm used for theconstruction of the OMM-1. The estimated set of deformation fields$\Psi ={\left\{\Psi_{T_{K}\to S_{j}}\right\}}_{j=1...J}$in OMM-1 space was used to model the average change inmorphology with age using Gaussian process (GP) regression (Rasmussen & Williams, 2005). The objectivecan be stated as finding the function that best models the change in brainmorphology as captured by the deformation fields given the subjects’agesx. The trained GP allowedthe prediction of a mean output deformation field$\tilde{\Psi }$in OMM-1 space for any (observed or unobserved) age$x_{*}$.Note that here we did not model differences in overall brain size and,consequently, the nonlinear deformation fields without their affinetransformation components were used.

Gaussian processes generalise the concept of Gaussian probabilitydistributions from stochastic variables to stochastic functions, and can bewritten as$\text{f}(\text{x})∼GP\left(m(\text{x}),\;\;k(\text{x},{\text{x}}^{\prime })\right)$ory=f(x)+ϵwith additive independent Gaussian noiseϵ.The GP is specified by its prior mean functionm(x), which is usually set to zero, and covariance function$k(\text{x},\;\;{\text{x}}^{\prime })$, whose form has to be manually chosen. Conceptually thesecan be seen as continuous generalisations of the mean vector and covariancematrix used to describe multivariate normal distributions of randomvariables. The joint distribution of the observed training input and outputpair (x,y) and unobserved pair($x_{*}$,$y_{*}$)can be written as

$$\left[\begin{array}{c} \text{y}\\ y_{*} \end{array}\right]∼N\left(0,\left[\begin{array}{cc} \text{K}\left(\text{x},\text{x}\right)+\sigma_{n}^{2}\text{I} & \text{k}\left(x,x_{*}\right)\\ \text{k}\left(x_{*},\text{x}\right) & k\left(x_{*},x_{*}\right) \end{array}\right]\right),$$ (7)

whereIis the identity matrixand$\sigma_{n}$describes the variance of the noise, with larger$\sigma_{n}$resulting in a smoother function.K(x,  x)is aJ×Jmatrix of covariances between all training inputs,$\text{k}(\text{x},\;\;x_{*})$and$\text{k}(x_{*},\;\;\text{x})$are vectors of covariances between training and queryinputs, and$k(x_{*},x_{*})$is the variance of the query input.

As will become more apparent fromEquations 10and11, the calculation of$\text{K}(\text{x},\text{x})+\sigma_{n}^{2}\text{I}$becomes increasingly computationally challenging withlargerJ.To ensure that all 37,330 individuals contribute to the GP training whilereducing the computational burden, the training input and output data werestratified along the age axis into half-yearly bins. Additionally, each binwas split into two sub-bins, where each of the 37,330 individuals wasrandomly assigned to one of two age-corresponding sub-bins per half-yearlybin, for example, two individuals of age 51.2 and 51.4 years would berandomly assigned to one of two sub-bins in the 51.0≤age<51.5 half-yearly bin. All individuals in a sub-bin were aggregated bycalculating their mean deformation field, leading to 148 sub-bins. Thisintroduction of some variability within each bin was done to bettercondition the estimation of the hyperparameters by making the estimates lesscorrelated. This aggregation considerably reduced the size of the trainingdataset from the initialJ=37,330data points toL=148$(2\;\text{sub-bins}\times 74\;\text{half-yearly mean agebins}\;\bar{\text{x}}\text{)}$and corresponding mean deformation fields$\bar{y}$as respective input and output for ages 45–81 years.

The noise term inEquation 7assumes that the noise is constant for every data point. This assumptionwould hold if every sub-bin was assigned the same number of individuals.However, when stratifying over age, the sub-bin averages were taken overdifferent number of individuals because of the nonuniform age distributionin the initial dataset—with fewer individuals for the youngest andoldest age groups. Assuming noise to be constant for all sub-bins wouldintroduce a bias. To account for this nonuniformity, the identity matrixIinEquation 7was replaced with aweight matrixWcontaining$\frac{1}{p_{l}}$in the diagonal, where$p_{l}$is the number of individuals assigned to age binl.This down-weighs the noise variance for, and increases the confidence in,bins pooled from a larger number of individuals, and vice versa. The jointdistribution fromEquation 7becomes

$$\left[\begin{array}{c} \bar{\text{y}}\\ y_{*} \end{array}\right]∼N\left(0,\left[\begin{array}{cc} \text{K}\left(\bar{\text{x}},\bar{\text{x}}\right)+\sigma_{n}^{2}\text{W} & \text{k}\left(\bar{\text{x}},x_{*}\right)\\ \text{k}\left(x_{*},\bar{\text{x}}\right) & k\left(x_{*},x_{*}\right) \end{array}\right]\right).$$ (8)

The choice of covariance function and its associated hyperparameters isimportant since it defines the properties of the functions generated duringinference. Here, a squared exponential kernel was used, which has strongsmoothness assumptions, and is, therefore, in line with the expected smoothchanges in the brain with age. The kernel for calculating the covariancebetween two agesxand$x^{\prime }$can be written as

$$k\left(x,x^{\prime }\right)=\sigma_{f}^{2}\text{exp}\left(-\frac{{\left(x-x^{\prime }\right)}^{2}}{2\ell^{2}}\right),$$ (9)

where$\sigma_{f}$is a scaling factor andℓis the length scale. Intuitively, a larger$\sigma_{f}$increases both the magnitude and the variability of the fitted function, anda larger length scaleℓincreases the dispersion and covariance between more distant ages, leadingto a smoother function, which is less influenced by noise andoverfitting.

The hyperparameters$\sigma_{f}$,$\sigma_{n}$,andℓcan be estimated by maximising the marginal likelihood given by

$$log\;p(\bar{\text{y}}|\sigma_{n},\sigma_{f},\ell )=-\frac{1}{2}{\bar{\text{y}}}^{\text{T}}{\left(\text{K}+\sigma_{n}^{2}\text{W}\right)}^{-1}\bar{\text{y}}-\frac{1}{2}log\left|\text{K}+\sigma_{n}^{2}\text{W}\right|$$, (10)

where$\bar{\text{y}}$is a matrix containing the vectorised deformation fields in the rows, andKis the covariancematrix where element$K_{ij}$is the covariance between two ages${\bar{x}}_{i}$and${\bar{x}}_{j}$.The Nelder–Mead simplex method has shown robust estimates whenminimising the negated function, which was optimised over 10,000 randomlyselected voxels within the brain to estimate the set of hyperparameters.

Using the estimated hyperparameters, the predictive mean can be calculatedwith

$$y_{*}\text{​}\;=k(x_{*},\;\bar{\text{x}}){\left(\text{K}(\bar{\text{x}},\;\bar{\text{x}})+\sigma_{n}^{2}\text{W}\right)}^{-1}\bar{\text{y}},$$ (11)

where the output$y_{*}$is the mean deformation field${\bar{\Psi }}_{x_{*}}$for the corresponding age$x_{*}$.One deformation field was predicted for each year in the age range45–81 years. The inverse of this deformation was concatenated withthe initial 240 subjects’ linear and nonlinear transformations suchthat

$$\Psi_{T_{x_{*}}\to R_{n}^{m}}={\bar{\Psi }}_{x_{*}}^{-1}\circ {\tilde{\Phi }}_{T_{k}\to R_{n}^{m}}$$ (12)

before resampling the corresponding modalities to age-specific template spacein one step. Averaging over each of the resampled modalities provided thecorresponding ADT$T_{x_{*}}$.

In contrast to GPs, we had also tested kernel regression for creating ADTs.However, applied to our data, kernel regression showed anatomically unstableresults, that is, brain regions in templates of consecutive years showedpronounced variability, which could be related to the manual choice ofhyperparameters such as the kernel width. We did not test GeneralizedAdditive Models for Location Scale and Shape (GAMLSS), since it can beassumed that the sampling variance is homogeneous over time. Hence, we donot think that the use of GAM would provide additional benefits over GPs,given their increased complexity.

### 2.5 Validation and applications

Convergence of the OMM-1 template construction process was assessed with threemetrics including the root mean squared (RMS) difference, the root mean squaredpercentage (RMSP) difference, and Pearson’s correlation (PC) betweenconsecutive iterations of average warps and T1, T2-FLAIR, and DTI volumes. TheFrobenius norm (FN) between consecutive iterations was additionally calculatedfor DTI volumes. Metrics based on the average warps show the improvement withrespect to the first objective function of finding the average shape template,while metrics based on the T1, T2-FLAIR, and DTI volumes show changes withrespect to the second objective function of finding the average intensitytemplate.

Our ADTs were visually assessed and the prediction-derived 81-year ADT wascompared to two directly estimated templates with the same age. The first ofthese two templates was constructed by registering all 101 UKB individuals inthe 80–81-year range (mean age of 80.44 years) to OMM-1 space. Theestimated deformation fields were spatially unbiased and applied to thecorresponding images, which allowed the construction of a directly estimatedADT-81. As a second template for comparison, we used the existing older adultMIITRA template (mean age of 80.56 years) (Wu etal., 2022). Due to the large age difference, we did not compare ourtemplate with the DTI atlas constructed from the Human Connectome Project (Irfanoglu et al., 2020). In addition to thepredicted ADTs within the training age range, we also predicted ADTs for ages20, 30, 40, 90, and 100 years to exemplify the GP’s characteristicbehaviour outside the training age range.

Age-related differences were assessed with the distortion given by averageJacobian determinant maps. One map was derived for each correspondingGP-predicted deformation field. Volume differences in the subcortical structureswere quantified by summing over corresponding ROIs in the Jacobian determinantmaps. The ROI masks were created by warping FSL FIRST (Patenaude et al., 2011) segmentation masks fromindividuals to OMM-1 space before averaging and binarising them with a thresholdof 0.5.

Finally, we investigated whether there is an advantage in registering individualsto the standard OMM-1 template via an age-matched ADT over registering themdirectly to the standard OMM-1. These results were also compared withregistering directly to the MNI 152 template. In total, 128 held-out UKB testsubjects (50% female) were uniformly sampled from the 50–81 years agerange of a different UKB release. Note that this release did not contain datafor the younger ages. The subjects were registered to (1) the OMM-1 directly,(2) the GP-derived ADT corresponding to the individual’s age, and (3) theMNI 152 directly. FSL FLIRT (Jenkinson &Smith, 2001) was used for all affine registrations followed by MMORF(Lange et al., 2024) for allnonlinear registrations. Registration parameters were identical for (1) and (2)with T1, T2-FLAIR, and DTI driving the nonlinear registrations, and T1-onlydriving the nonlinear registration for (3). DKT atlas ROIs (Desikan et al., 2006) had been created for eachindividual with FreeSurfer (Fischl et al.,2004) as part of the UKB preprocessing pipeline. These ROIs weretransformed from individuals’ native spaces to generic template spaceusing the direct warp to OMM-1 as estimated in (1), the composed warp fromindividual to ADT and from ADT to OMM-1 for (2), and the direct warp to MNI 152in (3) using trilinear interpolation. The Dice similarity coefficient for eachtransformed binarised ROI was calculated for every possible pairing of subjectsfor each of the three approaches. We repeated the same tests with 100out-of-sample individuals and their corresponding Destrieux atlas FreesurferROIs (Destrieux et al., 2010) from theHuman Connectome Project (HCP) (Van Essen etal., 2013) using T1-only, T1-only with brain extraction, and T1 andDTI modalities for registrations in (1) and (2). The 100 unrelated subjectssubset of the HCP young adult 1200 release (54 female, 46 male, mean age29.1±3.7years) was used as it provides a usefully large number ofsubjects but does not require correcting for family structure in group-levelanalyses (Hodge et al., 2016). Due to theyounger age range in the HCP, we used the youngest 45-year ADT as a referencespace for all registrations in (2).

## 3 Results

### 3.1 OMM-1

The template after the last iteration of each hierarchical level is shown inFigure 3A. Qualitatively, a gradualincrease in contrast and sharpness is noticeable in all three modalities withmore rapid change over the early iterations and less change in the lateriterations. Quantitative measurements of convergence towards the averageintensity and average shape are shown inFigure3B. The change in intensities and the change in average warps betweenconsecutive iterations show a similar pattern with all metrics. A sharp increasein difference occurs after switching to a finer registration level beforestabilising in the following iterations at the same level. These can be seen assharp spikes in the differences measured with RMS, RMSP, and FN, and sharp dropswith PC. These fluctuations become smaller in the later iterations, whichindicates convergence. For example, the PC similarity index for T1 templates ofconsecutive iterations at the last hierarchical level consistently reaches over0.99914. InAppendix A.4, we further showthe voxel-wise standard deviation across the resampled T1, T2-FLAIR, and FAimages for each of the last three iterations of the OMM-1 construction.

**Fig. 3.**
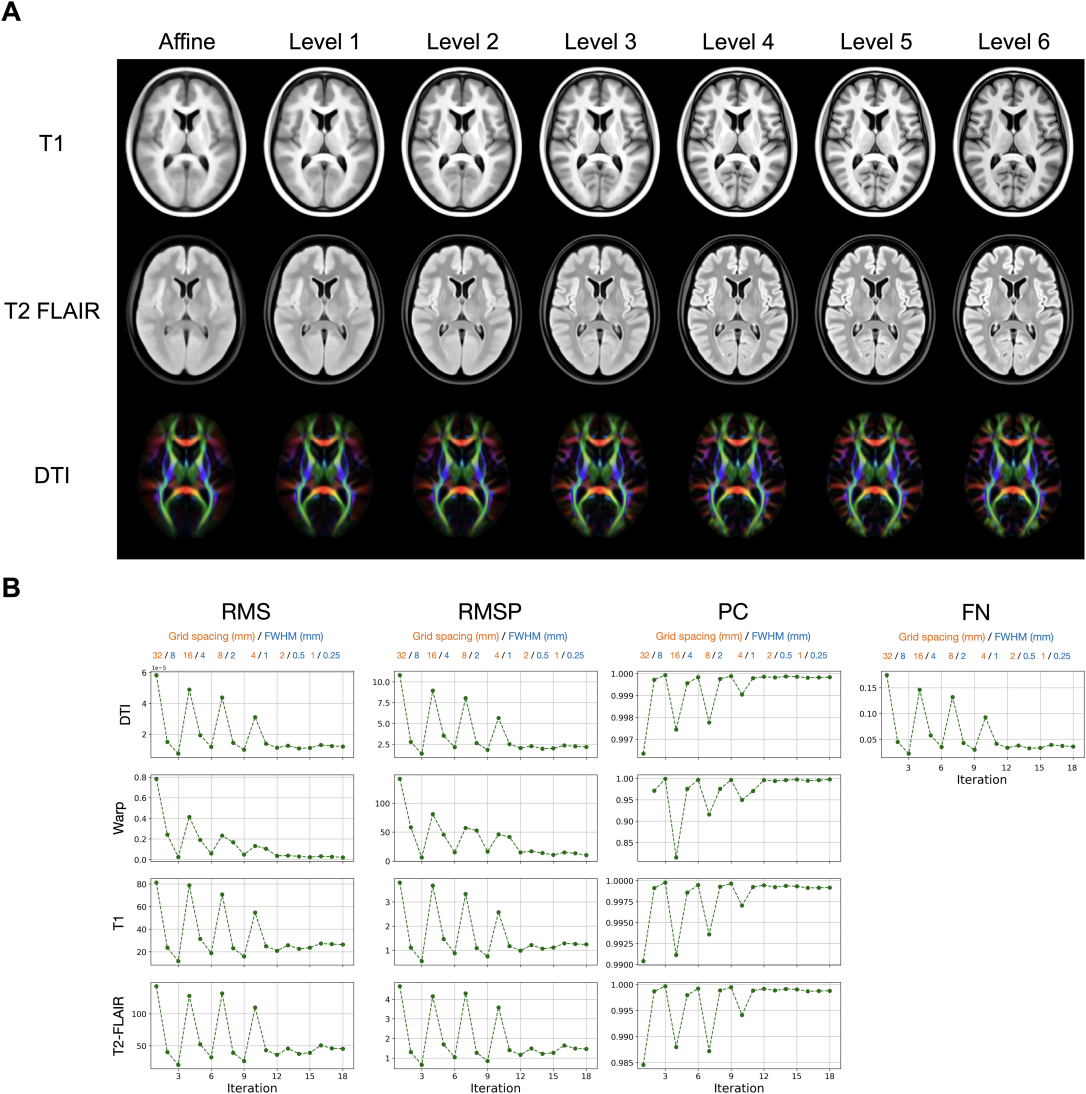
(A) Rows show the three template modalities: T1, T2-FLAIR, and DTI(visualised using a principal diffusion direction colour-coded FA mapwhere AP = green, LR = red, IS = blue). Theimprovement in contrast and alignment after the final iteration of eachhierarchical level is noticeable for each of the three modalities as thesize of the blurring kernel and the grid spacing are reduced from coarseto fine. (B) Convergence measured with several metrics—root meansquared (RMS), root mean squared percentage (RMSP), Pearson correlation(PC), and Frobenius norm (FN)—shows the difference betweentemplate intensities for each modality and between average warps ofconsecutive iterations. A sharp difference can be observed with allmetrics after switching to a new hierarchical level, which is followedby an improvement in the following iterations at the same level. Thesefluctuations stabilise towards the last iterations indicatingconvergence.

**Fig. 4.**
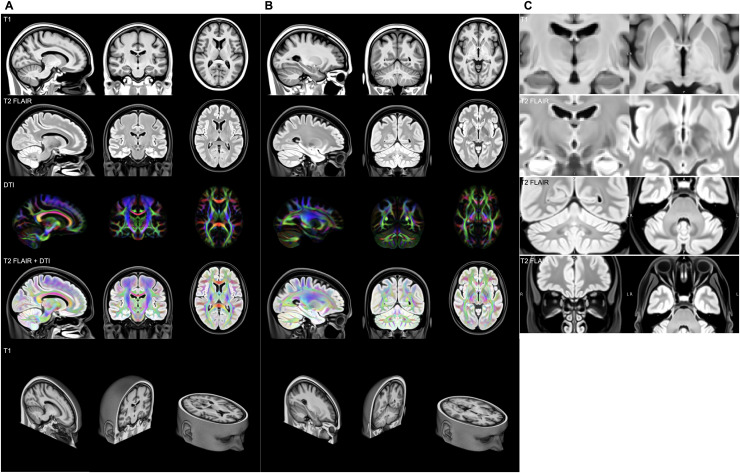
Two different sets of slices (A) and (B) through the modalities of thefinal OMM-1 template: T1, T2-FLAIR, DTI (visualised using a principaldiffusion direction colour-coded FA map where AP = green, LR= red, IS = blue), DTI overlaid on T2-FLAIR, and thecorresponding slice in the 3D T1 volume. Scalar T1 and T2-FLAIR volumesexhibit very good contrast. The DTI volume shows excellent sharpness andorientational consistency. Alignment between scalar and tensormodalities is exceptional as can be seen in the overlay of DTI andT2-FLAIR. Facial features such as the ears, nose, and eyes show a highlevel of detail. Coronal and axial slices show the right hemisphere onthe left and vice versa. Zoomed-in views of four ROIs in (C) highlightthe excellent alignment across all 240 participants. The medialmedullary lamina—separating internal from external globuspallidus—is clearly visible in the axial view of the T1 volume.On the T2-FLAIR volume, a clear separation of the subthalamic nucleusand substantia nigra can be seen in the coronal view, as well as thedentate nucleus in the cerebellum and the olfactory bulbs is clearlyvisible.

Two different sets of slices of the final OMM-1 template are shown inFigure 4. The T1 and T2-FLAIR volumes arevisually sharp with excellent contrast. In the DTI volume, white matter fibretracts appear clear and bright, and both isotropic and anisotropic regions arevery well aligned. This is especially noticeable in the cerebellum and thebrainstem tracts. All volumes, scalar, and DTI show very good alignment, asjudged by eye. This indicates that there are no systematic, rigid registrationerrors between modalities. Our strategy estimates a single warp from and appliesit to all modalities in individual spaces, which ensures correct cross-modalityalignment in template space (assuming that modalities in individual spaces arecorrectly aligned). Extracerebral structures, such as the sinuses, are clearlyvisible in the T2-FLAIR volume, as well as in the olfactory tract and theolfactory bulbs. Subcortical GM structures are sharply defined, withcomplementary contrast provided by the T1 and T2-FLAIR modalities (Fig. 4B).

### 3.2 Age-dependent templates

The prediction-based ADTs for selected ages in steps of 5 years are illustratedinFigure 5A. The scalar and tensor volumesshow consistently high quality of both contrast and crispness, and goodalignment between modalities. Expected age-related differences, such asincreases in ventricle sizes and sulcal widening, are visible in the templates,while the overall shape of anatomical brain structures and the folding patternremain stable across all ages. This is confirmed when looking at the Jacobiandeterminant maps, where the largest differences occur in the ventricles.Cortical GM thinning is noticeable in the insular cortex and the inferiorfrontal gyrus.

**Fig. 5.**
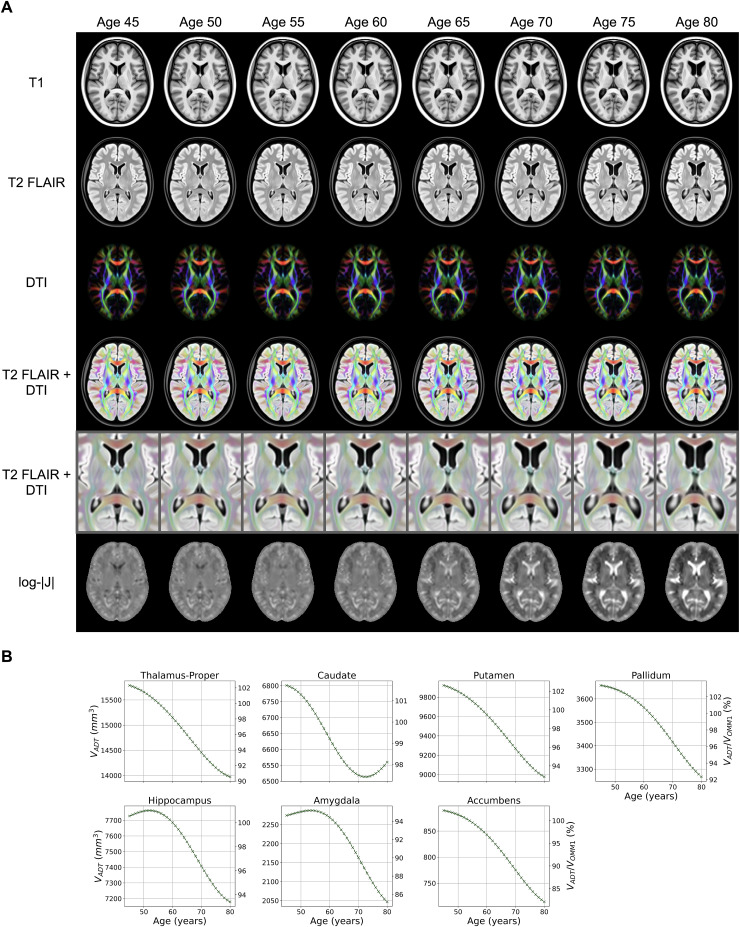
(A) The increase in ventricle size and sulcal widening can be observedacross all modalities in this subset of age-dependent templates (righthemisphere on the left). The expansion seen in ventricles and sulci andthe thinning of GM are visible in the corresponding log-Jacobian mapswith dark values indicating contraction and bright values indicatingexpansion of the main OMM-1 template. Thelog-|J|intensity range is set tolog(0.5)−log(2.0). (B) Volume measurements directly derived from thetemplates show characteristic age-related differences in subcorticalstructures. Absolute volumes are given on the left y-axis and percentvolume in comparison with OMM-1 on the right y-axis.

Age-related differences in volume can also be directly derived from the templatesas exemplified in subcortical structures inFigure5B, where an age-related loss in volume is noticeable for allstructures. A small unexpected increase in the hippocampus and amygdala volumeswas found before age 52 years. We further investigated whether this increase iscaused by an artefact of the modelling or the data by comparing measurementsfrom the GP-estimated 48-year Jacobian determinant map with those derived fromthe average Jacobian determinant map of all 45–50-year old individuals.We found that our model underestimated the relative volume by approximately 1.5%for the hippocampus and 1.1% for the amygdala in this age range, which will bepartly caused by the smaller number of individuals in this age range, leading tolarger uncertainty in the GP estimates. A similar pattern in the hippocampus wasobserved outside the main data range inJanahiet al. (2022), where GPs were directly applied to volumemeasurements. However, for the hippocampus, similar trajectories in volumedifference due to the data have been reported in the majority subgroup of thestudy population inFraser et al.(2021).

The increase in volume seen in the caudate from 72 years is an artefact caused bythe segmentation masks, where the increasingly large ventricles start bleedinginto the caudate ROI at older ages.

**Fig. 6.**
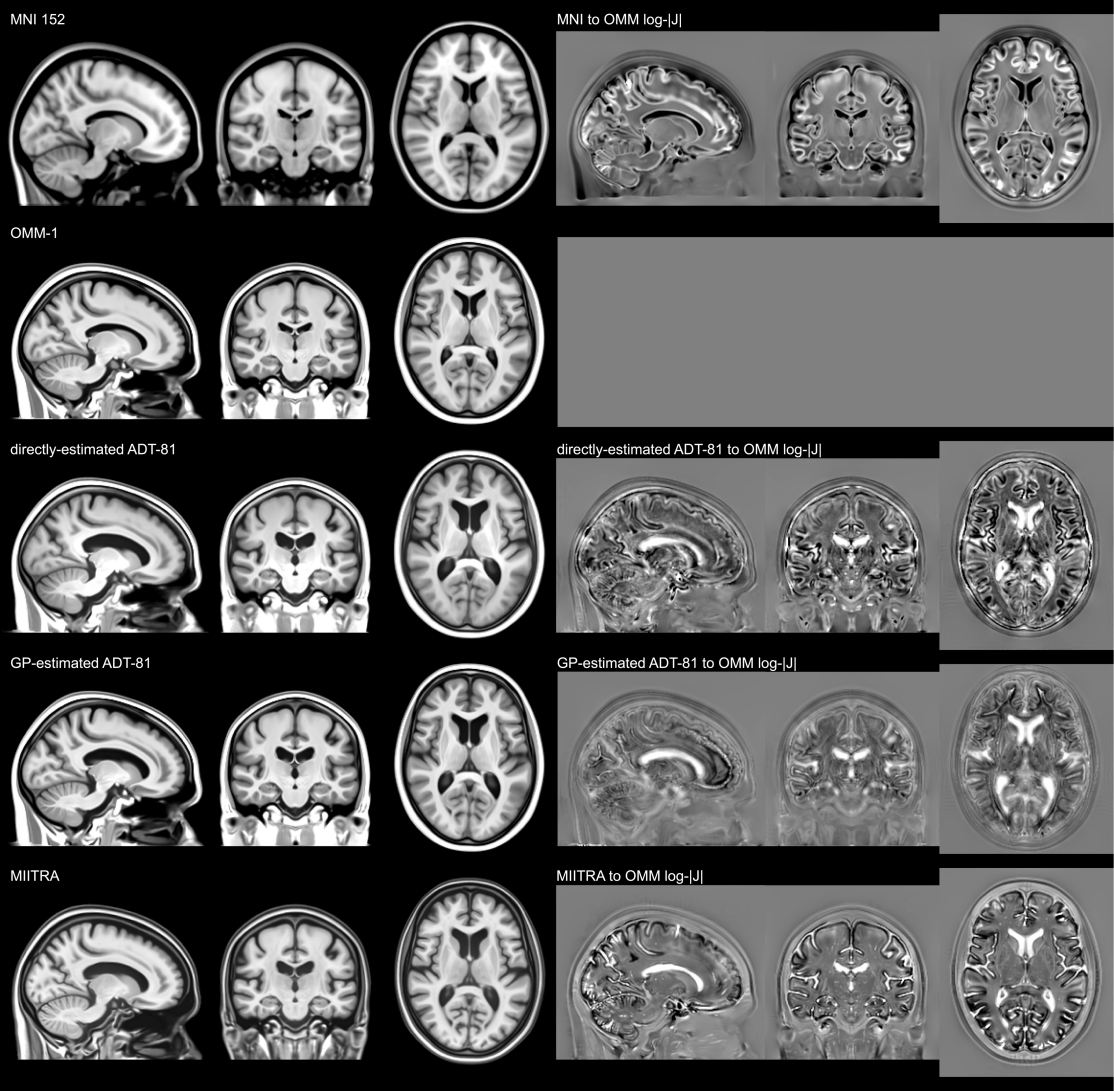
Visual comparison of the T1 volumes of the MNI 152, OMM-1, 81-year ADTdirectly estimated from 81-year old UKB individuals in OMM-1 space,81-year ADT estimated with the GP, and MIITRA template (rigidly aligned)(Wu et al., 2022). The rightcolumn shows the corresponding log-Jacobian maps (intensity range islog(0.5)–log(2)) of the registrations between the templates andthe OMM-1. The MNI 152 is provided as a reference and highlights thelarge global scale differences in MNI templates. Age-related differencebetween the OMM-1 and the two versions of the ADT-81 is well noticeable.The GP-estimated ADT-81 shows high similarity in overall shape andappearance, and age-related features such as ventricle size and sulcalwidening, with both the directly estimated ADT-81 and MIITRA. TheGP-estimated ADT was derived from the GP-transformed, original 240 OMM-1individuals (50–55-year age range). The directly estimated ADTand MIITRA were derived from older adults with mean ages of 80.44 yearsand 80.56 years, respectively.

Figure 6visually illustrates theage-related differences between the OMM-1 and the 81-year ADTs. It alsohighlights the high similarity between the directly estimated ADT, theGP-estimated ADT, and the MIITRA template (Wu etal., 2022). Note that the GP-estimated ADT was derived bytransforming the original 240 OMM-1 individuals from the 50–55-year agerange through a GP-predicted warp, while the directly estimated ADT was derivedby transforming and averaging individuals with a mean age of 80.44 years. MIITRAwas created through an iterative process from a cohort with a mean age of 80.56years. While the general shape of all three templates is very similar, there aresome differences. The directly estimated ADT has slightly larger ventricles thanboth the GP-estimated ADT and MIITRA. It is also slightly blurrier in corticalareas compared with the GP-estimated ADT. Our GP-estimated ADT shows improvedsharpness in subcortical areas, while MIITRA shows improved sharpness incortical areas such as the occipital lobe. The striped appearance of thestriatum is visible in the MIITRA, but not in the OMM-1 template.

The MNI 152 is provided as a commonly used reference template and highlights thelarge difference in scale compared with the OMM-1, ADT, and MIITRA templates.The cerebral volume of the OMM-1 is approximately 1,419,081 mm^3^,which is more than 465 ml less than the 1,884,594 mm^3^of the MNI 152and much more similar to the median and mean volumes of 1,433,335 mm^3^and 1,440,417 mm^3^, respectively, in the entire UKB. A checkerboardvisualisation inAppendix A.6allowsdirect visual comparison and further highlights the differences between OMM-1and MNI templates.

The log-Jacobian determinant maps corresponding to deformation fields predictedfor ages 20, 30, 40, 90, and 100 years, outside the training data range, areshown alongside a more detailed explanation of the GP’s behaviour inAppendix A.5.

### 3.3 Application to spatial normalisation

Relative differences in aggregated pairwise dice coefficients for each ROI on UKBdata are shown inFigure 7. Each dotrepresents for one ROI the relative difference between two aggregated overlapmeasurements, each achieved with a different template and each calculated as themean of the pairwise Dice coefficients. On average over all ROIs spatialalignment to the corresponding ADT slightly outperforms alignment to OMM-1 by0.5% in cortical and 0.28% in subcortical ROIs. Alignment to OMM-1 outperformsMNI 152 by a larger margin of 13.17% in cortical and 4.74% in subcortical ROIs.ADTs compared with OMM-1, and OMM-1 compared with MNI 152 achieve significantlylarger Dice overlaps in 49 and 51 out of 63 cortical ROIs, respectively. ADTscompared with OMM-1, and OMM-1 compared with MNI 152 achieve significantlylarger Dice overlaps in 22 and 23 out of 32 subcortical ROIs, respectively.

**Fig. 7.**
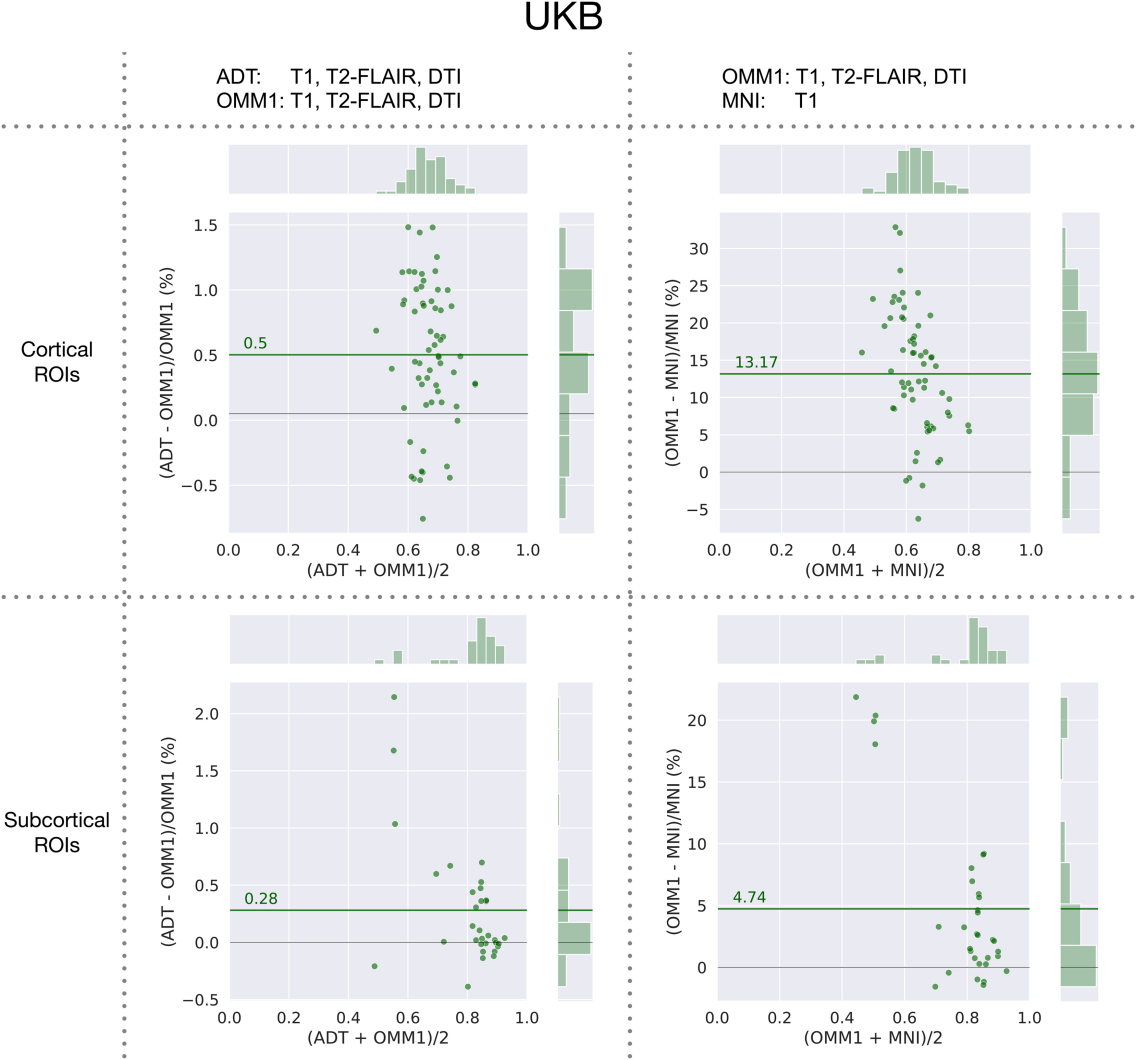
UK Biobank: relative differences between aggregated Dice coefficientsobtained by warping the same masks with transformations estimated whendirectly registering to OMM-1 (OMM1), indirectly registering to OMM-1via the individual’s corresponding age-dependent template (ADT),and registering to MNI 152. Each dot represents the relative differencefor one ROI and is calculated between the mean overlap values, that is,(mean of pairwise dice coefficients with one template—mean ofpairwise dice coefficients with second template)/(mean of pairwise dicecoefficients with second template). Results are shown separately forcortical ROIs (top row) and subcortical ROIs (bottom row). The greenline shows the average percentage difference over all respective ROIs.On average, ADTs slightly outperform OMM-1, and OMM-1 outperforms MNI152 by a larger margin.

**Fig. 8.**
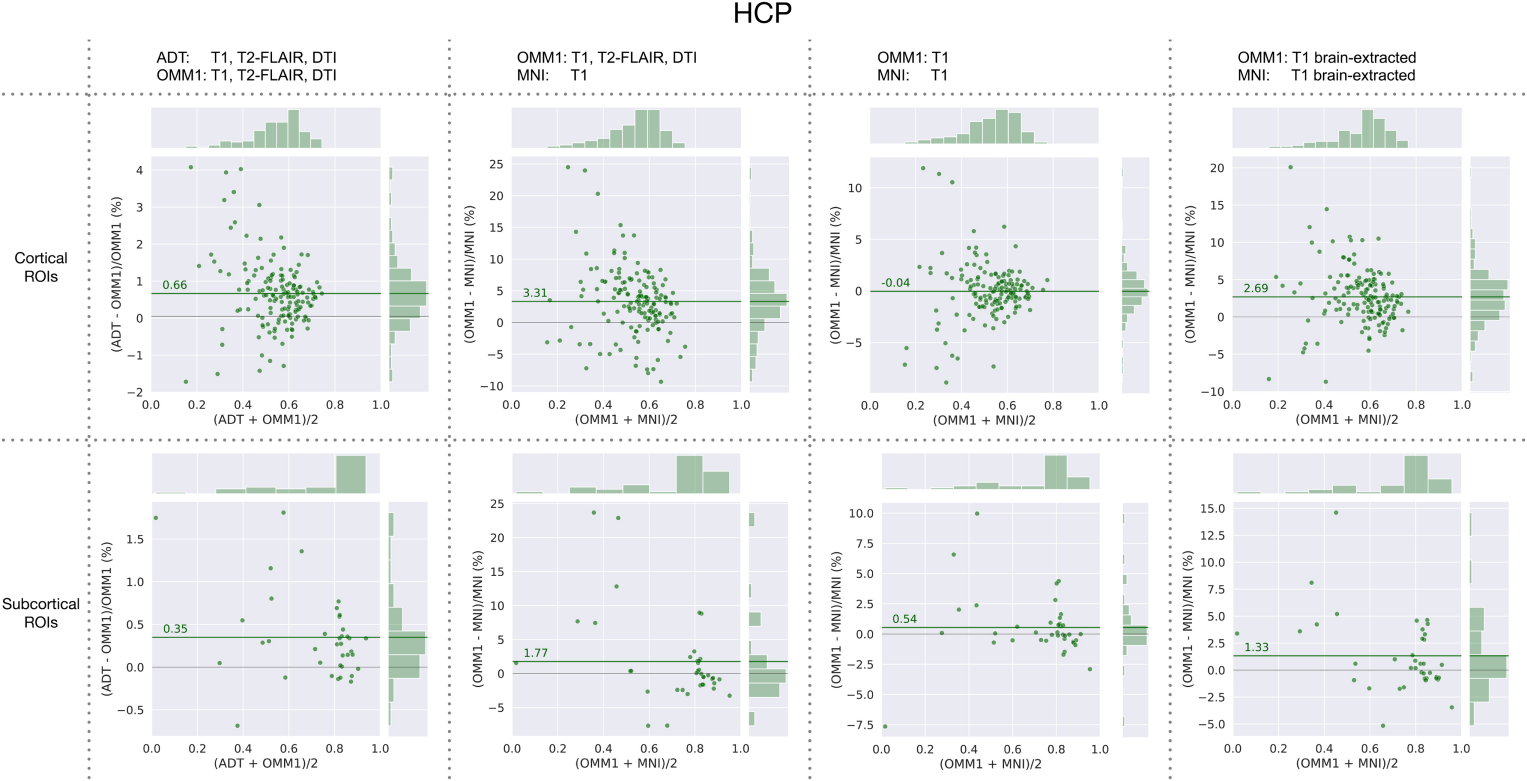
HCP: relative differences between Dice coefficients obtained by warpingthe same masks with transformations estimated when directly registeringto OMM-1 (T1, DTI), indirectly registering to OMM-1 via theindividual’s corresponding ADT, and registering to MNI 152.Additionally, registrations based on T1-only and brain-extractionT1-only were compared for OMM-1 and MNI 152. Each dot indicates one ROIand the green line shows the average percentage difference over allROIs.

Similar results can be replicated in an out-of-sample (non-UKB) cohort from theHCP (Fig. 8) where the 45-year ADToutperforms OMM-1 on average by 0.66% in cortical ROIs and 0.35% in subcorticalROIs. Multimodal registration to OMM-1 outperforms single-modal T1 registrationto MNI by 3.31% and 1.77%, respectively. Using only T1 for the registration toOMM-1 performs equally well as registration to MNI for cortical ROIs and 0.54%better for subcortical ROIs. Using preprocessed, brain-extracted images andtemplates for T1-based registrations showed a large improvement of OMM-1 overMNI 152 for both cortical and subcortical ROIs.

## 4 Discussion

We have presented the construction of the OMM-1, a fully unbiased, internallyconsistent, multimodal template of the brain including parts of the neck and face,averaging across 240 UKB individuals in the 50–55-year age range. GPregression was used to model deformation fields with age after registering 37,330UKB individuals to OMM-1, and allowed the prediction of an average warp for eachyear of age in the corresponding 45–81-year age range. These warp predictionswere used to resample the original 240 OMM-1 subjects and create one multimodal ADTfor each year of age. Test subjects from the UKB and HCP were registered to theOMM-1 directly, the ADT corresponding to the age of the individual, and MNI 152 tocompare their performance in spatial normalisation tasks.

OMM-1 and ADTs consist of T1, T2-FLAIR, and DTI volumes, and the same modalities wereused to jointly inform the template construction process through the use of MMORFfor all nonlinear registrations. The scalar volumes of all templates provideexcellent contrast and exceptional anatomical detail, and the DTI volume appearssharp in isotropic and anisotropic regions. Our strategy inherently provides optimalcross-modality alignment between template volumes since all modalities are resampledthrough the same warps.

The OMM-1 is rigidly (6 DOF) aligned to MNI 152 space to provide a basic level ofcomparability, while avoiding scaling effects, to preserve the average brain sizeand shape of the UKB population. The scaling factor (Jacobian determinant of theaffine transformation) between the OMM-1 and MNI 152 indicates an approximately 1.33times larger cerebral volume of the MNI 152, which is also visually noticeable inFigure 6. Although this difference in scalemight not have a large observable impact when used for spatial normalisation, it isnot optimal as it will require additional unnecessary distortion for the majority ofsubjects. However, we recognise that considerable effort has been put into thedevelopment of atlases and analysis of studies in MNI space, and we, therefore,provide a deformation field that maps between MNI and OMM-1 space to allow these tobe used with, or adapted for, our new template.

Note that approximately 93% of the randomly sampled 240 UKB individuals are of whiteethnicity, which is similar to the ethnic makeup of the entire UKB with 94% whiteparticipants and a general limitation of the dataset.

The GP model was used to create ADTs in steps of 1 year between ages 45 and 81 years,but allows for the construction of ADTs on a continuous scale within this age range.We would like to emphasize that the use of prediction-derived ADTs outside thetraining age range is not recommended. Extrapolation beyond this range will lead topredictions more similar to the prior mean function and, in turn the OMM-1 template,the more distant the age is to the training data. More information can be found inAppendix A.5.

Visual inspection (Fig. 6) shows that ourGP-estimated ADT-81 is highly similar in appearance to an ADT directly estimatedfrom 81-year UKB individuals. It also has highly similar shape- and age-relatedfeatures with respect to ventricle size, cortical folding, and global shape and sizecompared with the MIITRA template, which was directly constructed from an olderadult cohort. The slightly larger ventricles of the directly estimated ADT willlikely be caused by the smaller age range (80.0–81.0 years) of the subjectsused in its construction. This is in stark contrast to 65.2–94.9 years forMIITRA and the weighted contributions according to the GP hyperparameters for theGP-estimated ADT.

The GP-estimated ADT shows improved sharpness in subcortical areas, while MIITRAshows improved sharpness in cortical areas, especially in the occipital lobe.Improvements in the MIITRA template in these areas are likely due to their weightedaveraging approach, where intensities more similar to the median intensity acrosssubjects at a voxel location receive higher weights. Notably, the striped appearanceof the striatum is visible in the MIITRA, but not in the OMM-1. We have identifiedtwo reasonable causes for this. The first is that the regularisation metric used byMMORF to generate OMM-1, compared with that used by ANTs/DR-TAMAS to generateMIITRA, will more strongly penalise the deformations required to align the stripesacross individuals. Since MMORF optimises the structural and DTI alignmentsimultaneously, the deformations required to align the stripes would negativelyimpact the alignment of the tensors in that region (particularly in terms oforientation alignment). This is supported by experiments (not shown) where wegenerated templates using only the T1 channel, and in which the stripes were partlyvisible (but still not to the extent seen in MIITRA). The second is that there doesnot appear to be a clear biological indication on the consistency of the stripes(=pontes grisei caudatolentiformes alternating with white matter forming theinternal capsule) in number or exact location across individuals. Consequently, wedo not believe that this negatively affects the use of OMM-1 as a registrationtarget, even for older subjects.

We found that using the GP approach over simpler methods (such as kernel regressionor the direct estimation of a template for each year) produced ADTs where morphologynot affected by ageing (e.g., the folding pattern of the cortex) remained far morestable as a function of time. Our GP-based approach is also much more time efficientthan the repeated, direct construction of templates for specific ages. The mosttime-intensive tasks have to be performed only once, that is, the iterativeconstruction of a template (OMM-1) and the training of the GP. The estimatedhyperparameters can then be reused for the prediction of new templates thereafter.It is worth noting that the GP is fit to the deformation fields directly (i.e., weare applying algebraic operations, such as Euclidean averaging, as per the smalldeformation framework), which does not guarantee the preservation of diffeomorphism(Arsigny, Commowick, et al., 2006). Inthis instance, all estimated ADT warps did remain diffeomorphic (this is easy toverify empirically), but that should be explicitly checked whenever applying thisapproach to new data.

The loss of subcortical volume inFigure 5ismostly in line with results previously reported in the literature (Nobis et al., 2019;Vinkeet al., 2018;Wang et al., 2019;Walhovd et al., 2005). The increase inhippocampus and amygdala volumes for the younger ages was unexpected and appears tobe related to the smaller number of individuals available in this age range. This issimilar to the results obtained byJanahi et al.(2022), where a GP was applied to extracted volume measurements. Incomparison with direct measurements from the data, our model-derived ADTsunderestimate the volume by approximately 1.1 % to 1.5 % for this age range.However, it should be noted that a similar increase in hippocampus volume for thisage range has previously been reported (Fraser etal., 2021).

The increase in caudate volume seen from age 72 years was caused by the increasingsize of the ventricles at older ages bleeding into the caudate ROI. In addition toabsolute estimated volumes, we show relative volumes normalised by the volumesestimated in generic OMM-1 space. These ratios change at different rates fordifferent ROIs as would be expected. The volume change of the amygdala is a slightoutlier in that it does not reach 100%. The reason for both of these deviations isthe higher warp resolution of 1 mm used for the registrations in the construction ofthe OMM-1, compared with 2 mm for the GP-estimated ADTs. Increasing the warpresolution to 1 mm would produce more fine-grained average warps that might mitigatethis effect. However, given the increasingly large size of the dataset and thecorresponding number of required nonlinear registrations, 2 mm was found to be areasonable compromise, and is comparable with, or better than, the standard settingsof other registration methods.

The presence of pathologies such as white matter hyperintensities (WMH) ormicrobleeds at older ages has an impact on registration and subsequently onsegmentation accuracy in ADTs. Images of individuals in the 50–55-year agerange provide enhanced tissue contrast, more detail in anatomical structures, andless pathologies than images from older individuals. This enhanced quality was themain motivation behind choosing 240 younger subjects for the construction of theOMM-1, and is an advantage when using the templates for spatial normalisation, whichis generally the main use case for population-based templates. Common age-relatedpathologies not present in these 240 individuals will also not be present in ADTs,whose image intensities are all derived from the same 240 individuals. Similarly,DTI volumes of the ADTs will show expected differences in shape but not in thederived FA and MD maps. However, despite the use of these 240 younger individualsfor the GP-estimated ADTs, appearance did not show substantial morphologicaldifferences compared with the directly estimated ADT-81 (from 80–81-year oldUKB individuals) and the older adult MIITRA template. Although we did not seeevidence of WMHs adversely affecting the appearance of the older ADTs, cautionshould be taken when interpreting the ADT warps themselves in regions known tosystematically develop WMHs with ageing (e.g., periventricular WM). These 240individuals were taken from a sample that was filtered based on QC IDPs related tothe MNI template. While these IDPs can help identify individuals with pooralignment, or the presence of artefacts or outliers, they might also excludeanatomically difficult but otherwise normal individuals. Visual comparison of theOMM-1 template with the MIITRA template, which did not use these QC IDPs forfiltering, did not show signs of a bias due to these exclusions.

On average, alignment to OMM-1 via registration to an individual’sage-corresponding ADT shows slightly better spatial normalisation performance thanregistering directly to OMM-1 in both UKB and HCP test subjects. Although the MNI152 template is outside the age range of the UKB, it is commonly used for adultstudies of all ages and, as such, it was included in our comparison. The use ofOMM-1 and ADTs as template spaces outperformed MNI 152 on both UKB and HCP testsubjects for the majority of ROIs. The performance of MNI 152 was similar to T1-onlyregistration to OMM-1 when the skull was included. This can be explained by thelarge scalp signal present in the UKB T1 images and, consequently, also in OMM-1 andADTs that does not match the characteristics of HCP data. The sharp improvement ofT1-only registrations when used with a soft mask of the brain can be observed inFigure 8. Hence, we recommend the use of amask for the T1 template volume when used as registration reference in uni- andmultimodal registrations. It is also recommended to use a soft mask for DTI to avoidany potential impact of noisy tensors outside the brain. We supply such masks intemplate space as part of our OMM-1 release.

As a general note on using overlap measurements of automatically generatedsegmentations for validation, it should be added that, if these segmentations arerandomly unreliable, there are two sources of error—segmentation inaccuraciesand registration inaccuracies. If those errors are unrelated, then the overlapmetrics are affected by segmentation inaccuracies equally for each registrationmethod, that is, each choice of template. While this does not provide conclusiveevidence about the accuracy of registering to a single template, it does allow thecomparison of results across templates, since any improvements in performance willbe due to reduced registration inaccuracies and not due to differences insegmentation inaccuracies. If a registration template was biased in a way such thatit systematically tends to register images so as to match the segmentationinaccuracies, that method’s performance would be artificially inflated. SinceFreeSurfer and FIRST may use the MNI to aid in initialisation, such a situationcould favour the MNI templates in our comparison. However, since we do not see theMNI outperforming the OMM, we would expect that we are operating in a regime, wherethe segmentation and registration inaccuracies are uncorrelated.

Whether or not the benefits of slightly improved registration outweigh the addedcomplexity of using ADTs will likely depend on the specifics of a particular study.In many cases it may be sufficient to simply use the OMM-1 directly. However, shouldthe use of an ADT be preferred (e.g., the 80-year ADT for an older populationstudy), then our template provides a natural way to compare results with those fromother studies using the generic OMM-1, as well as making atlases defined in genericOMM-1 space available in any ADT. In addition to age, a new GP model could also beconditioned on other attributes such as sex. To get high confidence predictions,this approach requires datasets with sufficiently well-represented subpopulationswith the attribute of interest.

Our multimodal templating strategy provides a framework for integrating complementalinformation from scalar- and tensor-valued modalities with MMORF in a fully unbiasedand internally consistent way. The use of our templates in combination with MMORFcan largely improve accuracy in spatial normalisation tasks and the availability ofspatially corresponding information from anatomical images such as T1 and T2-FLAIR,and diffusion tensors from dMRI will greatly benefit the interpretation of resultsin template space. Our template construction pipeline is not limited to thesespecific modalities and can be readily applied to other modalities and datasets ofinterest. In the future, we hope to further extend the field of view of the OMM-1 toinclude the whole neck and face, which could add further benefits for MEG and EEGstudies.

## Figures and Tables

**Appendix Fig. A.1.**
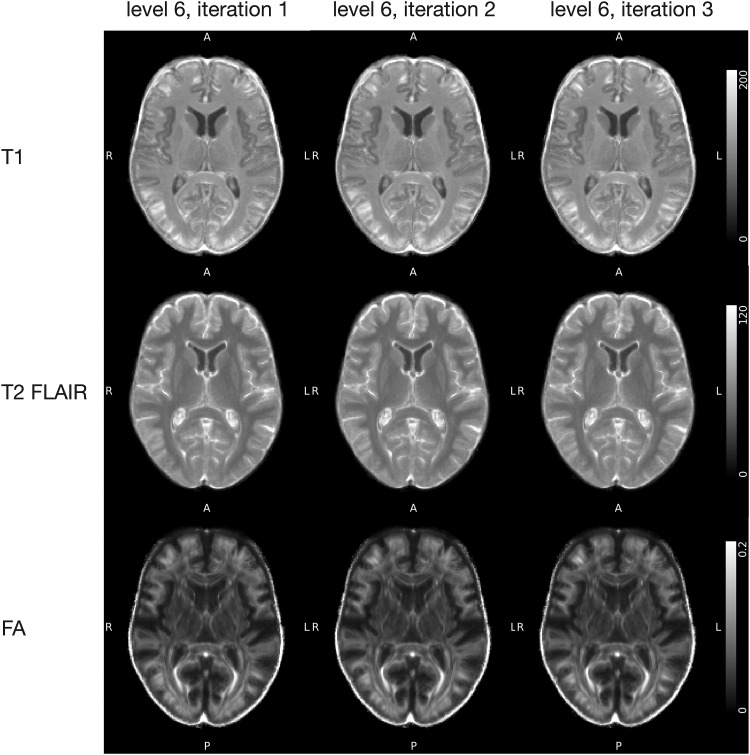
The same slice through the T1, T2-FLAIR, and FA images showing thevoxel-wise standard deviation of the resampled images for each ofthe last iterations of the OMM-1 template construction process.

**Appendix Fig. A.2.**
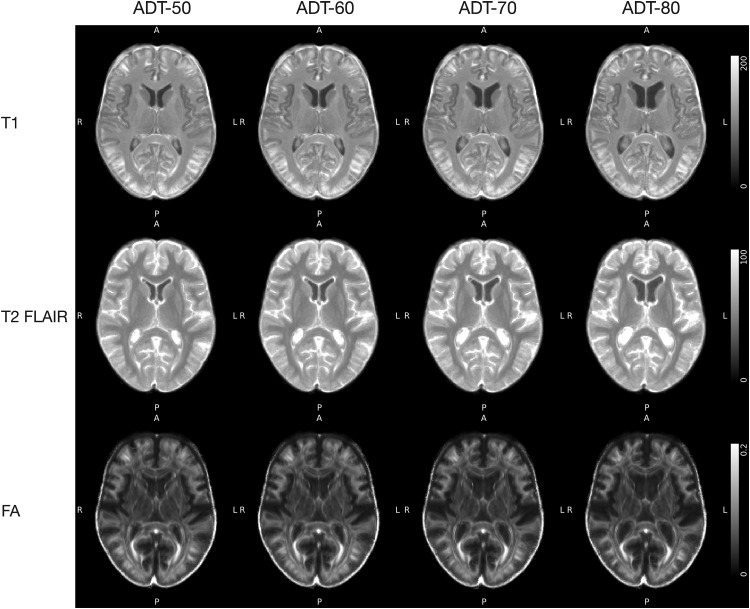
The same slice through the T1, T2-FLAIR, and FA images showing thevoxel-wise standard deviation of the resampled images for ADTs withages 50, 60, 70, and 80 years.

**Appendix Fig. A.3.**
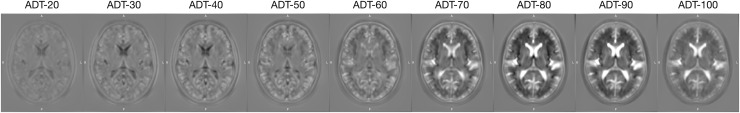
The same slice through the log-Jacobian determinant mapscorresponding to ADTs predicted within and outside the training agerange. The intensity scale is in the range oflog(0.5)–log(2).

**Appendix Fig. A.4.**
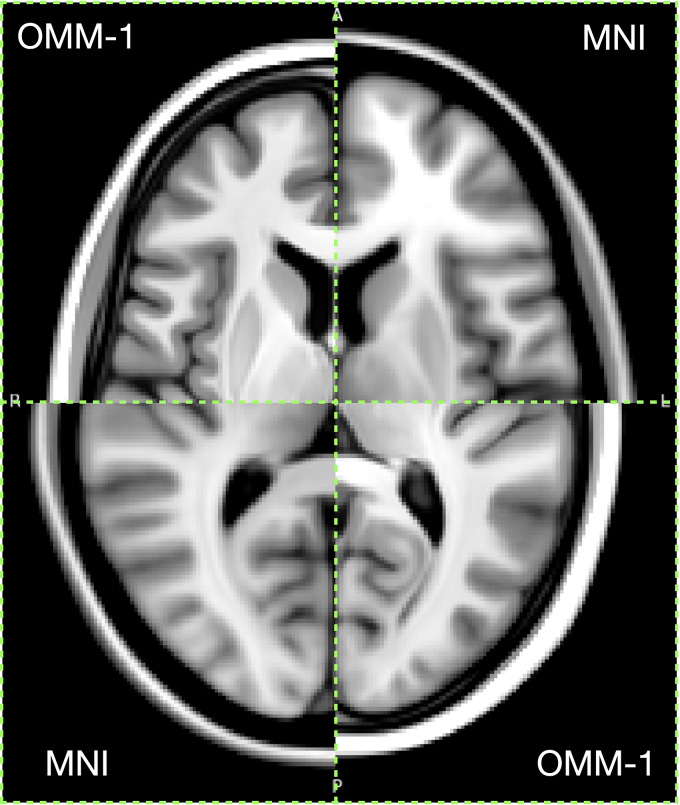
The larger scale and reduced sharpness of the MNI template comparedwith the OMM-1 can be visually highlighted when comparing themdirectly.

**Appendix Fig. A.5.**
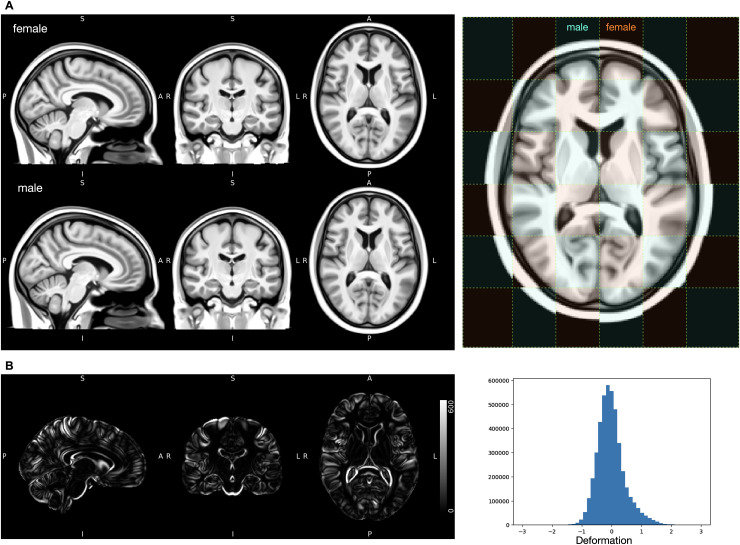
(A) Female and male templates after removing affine differences. Avisual comparison shows only small differences in shape. (B) This isalso demonstrated by the difference image and the histogram of thedeformation within the brain of the male-to-female warp.

## Data Availability

OMM-1 and all preconstructed ADTs, the code for the construction of a template, andthe MMORF config files used for the nonlinear registrations can be publicly accessedviahttps://osf.io/s9ge4(Arthofer et al., 2023). The code for theconstruction of a multimodal template^†^and relevant code for ADT construction^‡^can also be directly accessed.We used Python 3.7, FSL version 6.0.5, fslpy version 3.7.0, and MMORF version 0.2.4.The publicly available code for generating templates was tested with Python 3.9, FSLversion 6.0.7, fslpy version 3.13.0, and MMORF version 0.3.2.

## Footnotes

* Only the b = 1000 shell is used for tensor fitting due to the violation ofthe Gaussian diffusion assumption underlying the diffusion tensor model athigher b-values (or when combining b-values), which would require Kurtosis forcorrect modelling.

## Appendix A

### Appendix A.1. Dealing with high-intensity values

Clamping of high scalp intensities was achieved with a custom sigmoidclamping function

$$f(x)=\{\begin{array}{c} 0\\ x\\ k/\left(2*\left(1+exp\left(-8*(x-k)\text{​}/k\right)\right)\right)+0.75*k \end{array}\begin{array}{cc} & \text{if }x\leq 0\\ & \text{if}\;0<x\leq k\\ & \text{if }x>k \end{array}$$
(A.1)

wherexis a voxel’s intensity andkthe mean WM intensity.

### Appendix A.2. Gaussian Process implementation

The GP was reimplemented based on our own code to provide more flexibilitywith different optimisation methods and parts of existing code from thescikit-learn implementation (Pedregosa etal., 2011). We tested three different types of optimisation basedon log-marginal likelihood, cross-validation, and Geisser’spredictive probability. An overview of the methods can be found inSundararajan and Keerthi (2001).Log-marginal-likelihood showed the most stable behaviour and was used forthe final ADTs. The code of the implementation can be found in addition tothe ADTs on GitLab.^§^

### Appendix A.3. MMORF registration parameters

Appendix Table A.1contains theMMORF registration parameters used for the construction of the OMM-1 andage-dependent templates. The main difference is in the maximum warpresolution which is 1 mm for the former and 2 mm for the latter. The choiceof reducing the warp resolution from 1 to 2 mm was mainly motivated bysavings in runtime, while still maintaining a high level of detail.

**Appendix Table A.1:** MMORF parameters used for the registrations when constructing theOMM-1 and age-dependent templates.

	OMM-1	Age-dependent templates
; general		
warp_res_init	32	32
warp_scaling	1 1 2 2 2 2 2	1 1 2 2 2 2
lambda_reg	4.0e5 3.7e-1 3.1e-1 2.6e-1 2.2e-1 1.8e-1 1.5e-1	4.0e5 3.7e-1 3.1e-1 2.6e-1 2.2e-1 1.8e-1
hires	3.9	3.9
optimiser_max_it_lowres	10	5
optimiser_max_it_hires	10	5
; whole head T1		
use_implicit_mask	0	0
use_mask_ref_scalar	1 1 1 1 1 1 1	1 1 1 1 1 1 1
use_mask_mov_scalar	0 0 0 0 0 0 0	0 0 0 0 0 0
fwhm_ref_scalar	8.0 8.0 4.0 2.0 1.0 0.5 0.25	8.0 8.0 4.0 2.0 1.0 0.5
fwhm_mov_scalar	8.0 8.0 4.0 2.0 1.0 0.5 0.25	8.0 8.0 4.0 2.0 1.0 0.5
lambda_scalar	1 1 1 1 1 1 1 1	1 1 1 1 1 1
estimate_bias	1	1
bias_res_init	32	32
lambda_bias_reg	1e9 1e9 1e9 1e9 1e9 1e9 1e9	1e9 1e9 1e9 1e9 1e9 1e9
; whole head FLAIR		
use_implicit_mask	0	0
use_mask_ref_scalar	0 0 0 0 0 0 0	0 0 0 0 0 0
use_mask_mov_scalar	0 0 0 0 0 0 0	0 0 0 0 0 0
fwhm_ref_scalar	8.0 8.0 4.0 2.0 1.0 0.5 0.25	8.0 8.0 4.0 2.0 1.0 0.5
fwhm_mov_scalar	8.0 8.0 4.0 2.0 1.0 0.5 0.25	8.0 8.0 4.0 2.0 1.0 0.5
lambda_scalar	1 1 1 1 1 1 1	1 1 1 1 1 1
estimate_bias	1	1
bias_res_init	32	32
lambda_bias_reg	1e9 1e9 1e9 1e9 1e9 1e9 1e9	1e9 1e9 1e9 1e9 1e9 1e9
; DTI		
use_mask_ref_tensor	1 1 1 1 1 1 1	1 1 1 1 1 1
use_mask_mov_tensor	1 1 1 1 1 1 1	1 1 1 1 1 1
fwhm_ref_tensor	8.0 8.0 4.0 2.0 1.0 0.5 0.25	8.0 8.0 4.0 2.0 1.0 0.5
fwhm_mov_tensor	8.0 8.0 4.0 2.0 1.0 0.5 0.25	8.0 8.0 4.0 2.0 1.0 0.5
lambda_tensor	1 1 1 1 1 1 1	1 1 1 1 1 1

Given the large number of individuals used in the age-dependenttemplate modelling and the large associated computationalrequirements, the highest warp resolution was set to 2 mm, incontrast to 1 mm used for the OMM-1.

### Appendix A.4. Voxel-wise variation at the end of template construction

Appendix Figures A.1andA.2show the voxel-wise standarddeviation of the resampled images for the last iterations of the OMM-1construction process and ADTs with different ages. A similar pattern isnoticeable for all templates where larger standard deviation can be observedin the occipital and frontal parts of the brain. This will be partly causedby remaining morphological variability, intensity differences, and theregistration taking into account other modalities. Note that due to themultimodal nature of our templates, the voxel-wise variation within a singlemodality might not be most representative when trying to capture the levelof convergence.

### Appendix A.5. ADT prediction outside training data range

A GP is defined through its prior mean function and covariance function,where the mean function is commonly assumed to be zero. In our case, thiswould be a deformation field with zero displacement, which, in turn, wouldproduce an ADT identical to the OMM-1. When making predictions outside afitted data range, GPs would be expected to smoothly change theirpredictions back towards this prior mean function. Consequently, our modelwould produce ADTs more and more similar to OMM-1 the more distant theprediction age is from the fitted age range. We can demonstrate thesetheoretical characteristics of GPs by predicting deformation fields outsidethe age range. We estimated ADTs for ages 20, 30, 40, 90, and 100 yearsoutside the existing data age range in addition to ADTs for ages 50, 60, 70,and 80 years from within the age range.Appendix Figure A.3shows a comparison of log-Jacobiandeterminants corresponding to the estimated deformation fields.

The GP extrapolates beyond the data age range continuing with a similartrend, as can be exemplified on the ADT-40, where the ventricles requirestronger compression than in the ADT-50, that is, a 40-year-old individualwould on average have smaller ventricles than a 50-year-old individual.However, in the ADT-30, this trend has reversed, that is, values throughoutthe whole brain go towards 1 (no change). A similar behaviour can be seen atthe opposite end of the age spectrum, where the expansion of the ventriclescontinues beyond the existing data range, for example, ADT-90 shows moreexpansion than ADT-80, but values across the whole brain in the ADT-100 gotowards 1 (no change).

### Appendix A.6. Further comparison of the OMM-1 and MNI template

The checkerboard visualisation of the OMM-1 and MNI templates inAppendix Figure A.4further highlightstheir differences in scale and sharpness (OMM-1 (top-left, bottom-right),MNI (top-right, bottom-left)).

### Appendix A.7. Further comparison of the female and male templates

We have also created female and male templates by using a similar unbiasingapproach to the one used for ADT construction. We averaged the warps fromthe 120 females and applied the inverse of the average warp to the OMM-1template. The same procedure was repeated for the warps from the 120 males.Affine differences in brain size were removed by the initial affinetransformations. The resulting male and female templates are shown inAppendix Figure A.5A. A visualassessment of the brains and the checkerboard visualisation did not shownoticeable differences in shape, which was also demonstrated by thedifference image and the histogram of the male-to-female warp, that is, thedeformation inside the brain (Appendix Fig.A.5B).
